# Supplementary material for: Antimicrobial and Cytotoxic Activities of Constituents from the Fruit of Albizia lebbeck L. Benth (Fabaceae)
Source: Molecules. 2022 Jul 28;27(15):4823. doi: 10.3390/molecules27154823 (PMC9369593; doi:10.3390/molecules27154823)
Supplement: Supplementary file 1 [file molecules-27-04823-s001.zip › molecules-1813330-supplementary.pdf]

Antimicrobial and cytotoxic activities of constituents from the fruits of *Albizia lebbeck* L. Benth (Fabaceae)

Bosco Peron Leutcha[1,2], Jean Paul Dzoyem[3], Jean-Bosco Jouda[4], Denis Kehdinga Sema[1], Virginie Flaure Tsague Tankeu[1], Gabin Thierry M'bahbou Bitchagno[5,6], Billy Toussie Tchegnitegni[2,5,6], Flaure Rosette Ehawa Essoung[7], Bruno Ndjakou Lenta[2], Siméon Fogue Kouam[2], Alain Meli Lannang[4,8,*], Norbert Sewald[6,*].

[1]Department of Chemistry, Faculty of Science, University of Maroua, P.O. Box 814, Maroua, Cameroon; peron.leutcha@gmail.com (B.P.L); devansema@gmail.com (D.K.S.); vtsague10@gmail.com (V.F.T.T);

[2]Department of Chemistry, Higher Teacher Training College, University of Yaoundé I, P.O. Box 47, Yaoundé, Cameroon; peron.leutcha@gmail.com (B.P.L); billytoussie@yahoo.fr (B.T.T.); lentabruno@yahoo.fr (B.N.L.); kfogue@yahoo.com (S.F.K.)

[3]Department of Biochemistry, Faculty of Science, University of Dschang, P.O. Box 67, Dschang, Cameroon; jpdzoyem@yahoo.fr (J.P.D)

[4]Department of Chemical Engineering, School of Chemical Engineering and Mineral Industries, P.O. Box 454, Ngaoundéré, Cameroon; joudabosco@yahoo.fr (J-B.J); alainmeli@yahoo.com (A.M.L.)

[5]Departement of Chemistry, Faculty of Science, University of Dschang, P.O. Box 67, Dschang, Cameroon; bmgt198716@ymail.com (G.T.M.B); billytoussie@yahoo.fr (B.T.T)

[6]Department of Chemistry, Organic and Bioorganic Chemistry, Bielefeld University, 33501 Bielefeld, Germany; bmgt198716@ymail.com (G.T.M.B); billytoussie@yahoo.fr (B.T.T.); nobert.sewald@uni-bielefeld.de (N.S.)

[7]University Institute of Technology, University of Ngaoundere, P.O. Box 455, Ngaoundere, Cameroon; rosflaure@yahoo.fr (F.R.E.E.)

[*] Correspondance:about:blank alainmeli@yahoo.com

[*] Correspondance:about:blank nobert.sewald@uni-bielefeld.de

[8]Department of Chemistry, Higher Teacher Training College, University of Maroua, P.O. Box 55, Maroua, Cameroon; alainmeli@yahoo.com (A.M.L.)

Table of Contents

Figure S1. HR-ESI-MS (+) spectra of compound **1**

Figure S2. ^{1}H-NMR spectrum (D_2O, 600 MHz) of compound **1**

Figure S3. ^{13}C-NMR spectrum (D_2O, 150 MHz) of compound **1**

Figure S4. HSQC spectrum of compound **1**

Figure S5. COSY ^{1}H-^{1}H spectrum of compound **1**

Figure S6. HMBC spectrum of compound **1**

Figure S7. NOESY spectrum of compound **1**

Figure S8. HR-ESI-MS (+) spectrum of compound **2**

Figure S9. ^{1}H-NMR spectra (600 MHz, DMSO-d_6) of compound **2**

Figure S10. ^{13}C-NMR and DEPT-135 spectra (150 MHz, DMSO- d_6) of compound **2**

Figure S11. HMQC spectrum of compound **2**

Figure S12. COSY 1H-1H spectrum of compound **2**

Figure S13. HMBC spectrum of compound **2**

Figure S14. NOESY spectrum of compound **2**

Figure S15. ^{1}H-NMR spectrum (500 MHz, MeOD) of compound **3**

Figure S16. ^{13}C-NMR and DEPT-135 spectra (125 MHz, MeOD) of compound **3**

Figure S17. ^{1}H-NMR spectrum (500 MHz, MeOD) of compound **4**

Figure S18. ^{13}C-NMR and DEPT135 spectra (125 MHz, MeOD) of compound **4**

Figure S19. ^{1}H-NMR spectrum (MeOD, 500 MHz) of compound **5**

Figure S20. ^{13}C-NMR and DEPT-135 spectra (MeOD, 125 MHz) of compound **5**

Figure S21. ^{1}H-NMR spectrum ($CDCl_3$; 500 MHz) of compounds **6** and **7**

Figure S22. ^{13}C-NMR and DEPT135 spectra ($CDCl_3$; 125 MHz) of compounds **6** and **7**

Figure S23. ^{1}H-NMR spectrum ($CDCl_3$, 500 MHz) of compound **8**

Figure S24. ^{13}C-NMR &DEPT-135 spectra ($CDCl_3$, 125 MHz) of compound **8**

Figure S25. ^{1}H-NMR spectrum ($CDCl_3$, 400 MHz) of compound **9**

Figure S26. ^{1}H-NMR spectrum ($CDCl_3$, 500 MHz) of compound **10**

Figure S27. ^{13}C-NMR and DEPT-135 spectra ($CDCl_3$, 125 MHz) of compound **10**

Figure S28. ^{1}H-NMR spectrum (MeOD; 600 MHz) of compound **11**

Figure S29. ^{13}C-NMR and DEPT-135 spectra (MeOD; 150 MHz) of compound **11**

Figure S30. ^{1}H-NMR spectrum (D_2O, 600 MHz) of compound **12**

Figure S31. ^{13}C-NMR spectrum (D_2O, 150 MHz) of compound **12**

Figure S32. ^{1}H-NMR spectrum (DMSO-d_6, 600 MHz) of compound **13**

Figure S33. ^{13}C-NMR spectrum (DMSO-d_6, 150 MHz) of compound **13**

Figure S34. ^{1}H-NMR spectrum ($CDCl_3$, 500 MHz) of compound **14** and **16**

Figure S35. ^{1}H-NMR spectrum (DMSO-d_6, 500 MHz) of compounds **15** and **17**

Figure S36. ^{13}C-NMR spectrum (DMSO-d_6, 125 MHz) of compound **17**

Figure S37. ^{1}H-NMR spectrum ($CDCl_3$, 500 MHz) of compound **18**

Figure S38. ^{1}H-NMR spectrum (MeOD, 500 MHz) of compound **19**

Figure S39. ^{13}C-NMR spectrum ($CDCl_3$/MeOD, 125 MHz) of compound **19**

Figure S40. ^{1}H-NMR spectrum (DMSO-d_6, 500 MHz) of compound **20**

Figure S41: ^{13}C-NMR spectrum (DMSO-d_6, 500 MHz) of compound **20**

Figure S42. ^{1}H-NMR spectrum (Pyridine-d_5, 500 MHz) of compound **21**

Figure S43. ^{13}C-NMR spectrum (Pyridine-d_5, 125 MHz) of compound **21**

Figure S44. ^{1}H-NMR spectrum (DMSO-d_6, 500 MHz) of compound **22**

Figure S45. ^{13}C-NMR and DEPT-135 spectra (DMSO-d_6, 125 MHz) of compound **22**

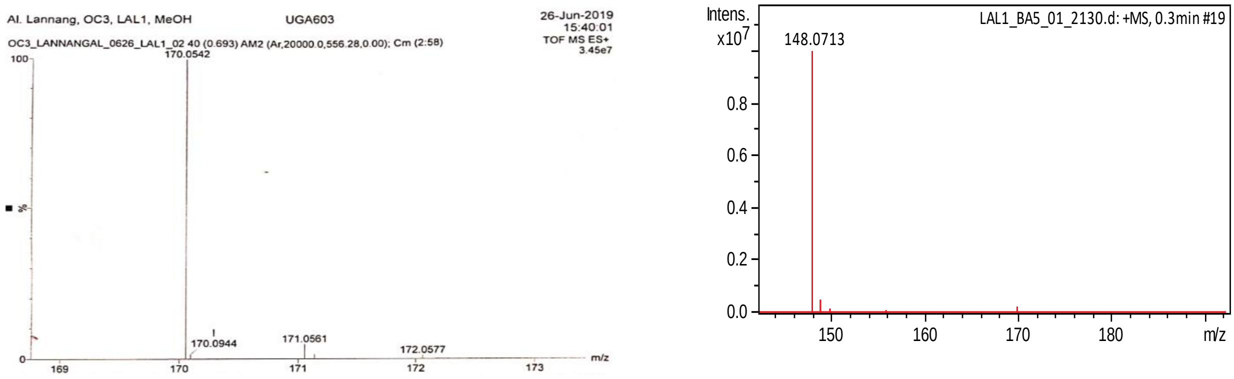

[M+Na]$^+$ **[M+H]$^+$**

Figure S1. HR-ESI-MS (+) spectra of compound **1**

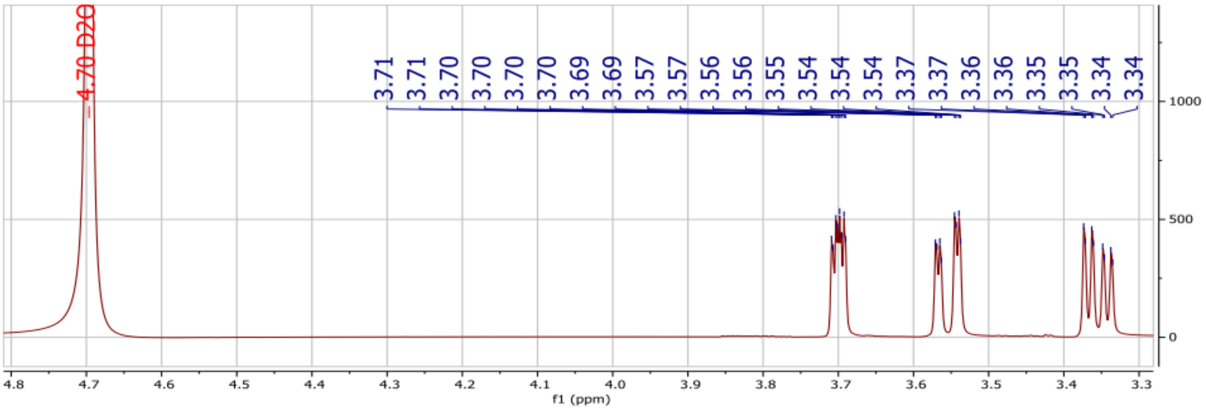

Figure S2. ^{1}H-NMR spectrum (D_2O, 600 MHz) of compound **1**

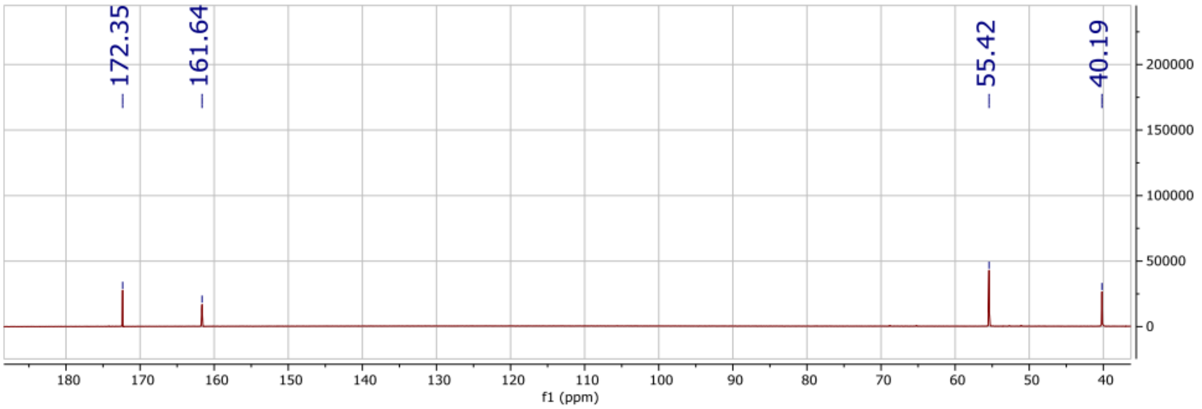

Figure S3. ^{13}C-NMR spectrum (D_2O, 150 MHz) of compound **1**

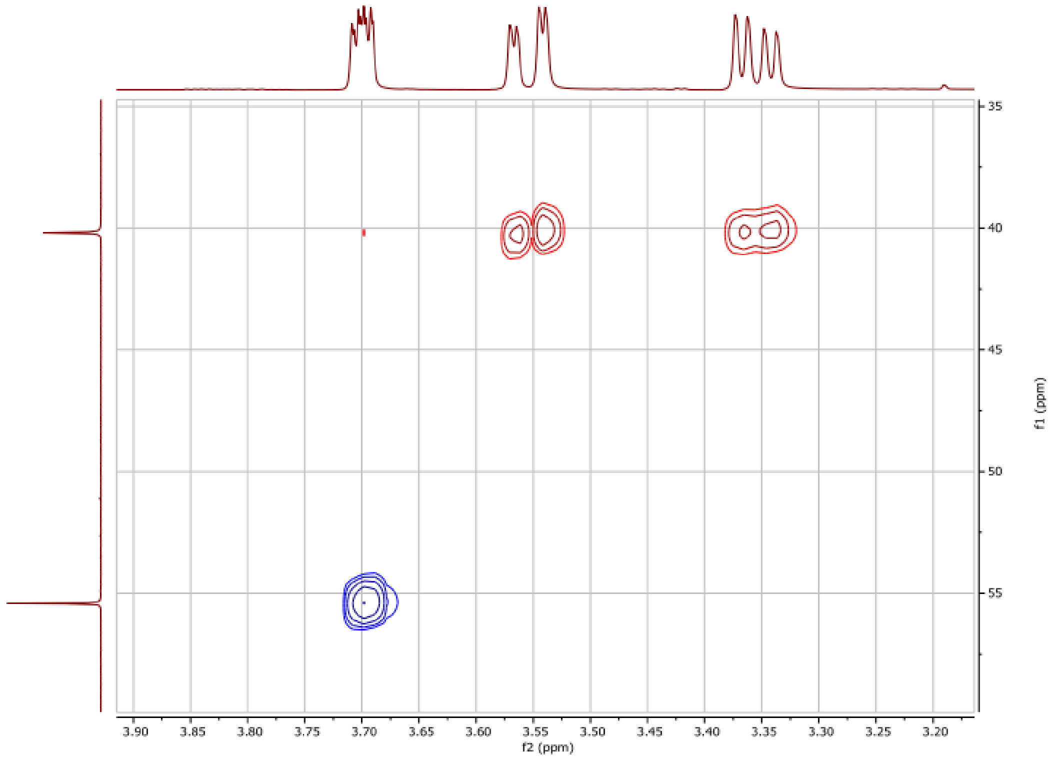

Figure S4. HSQC spectrum of compound **1**

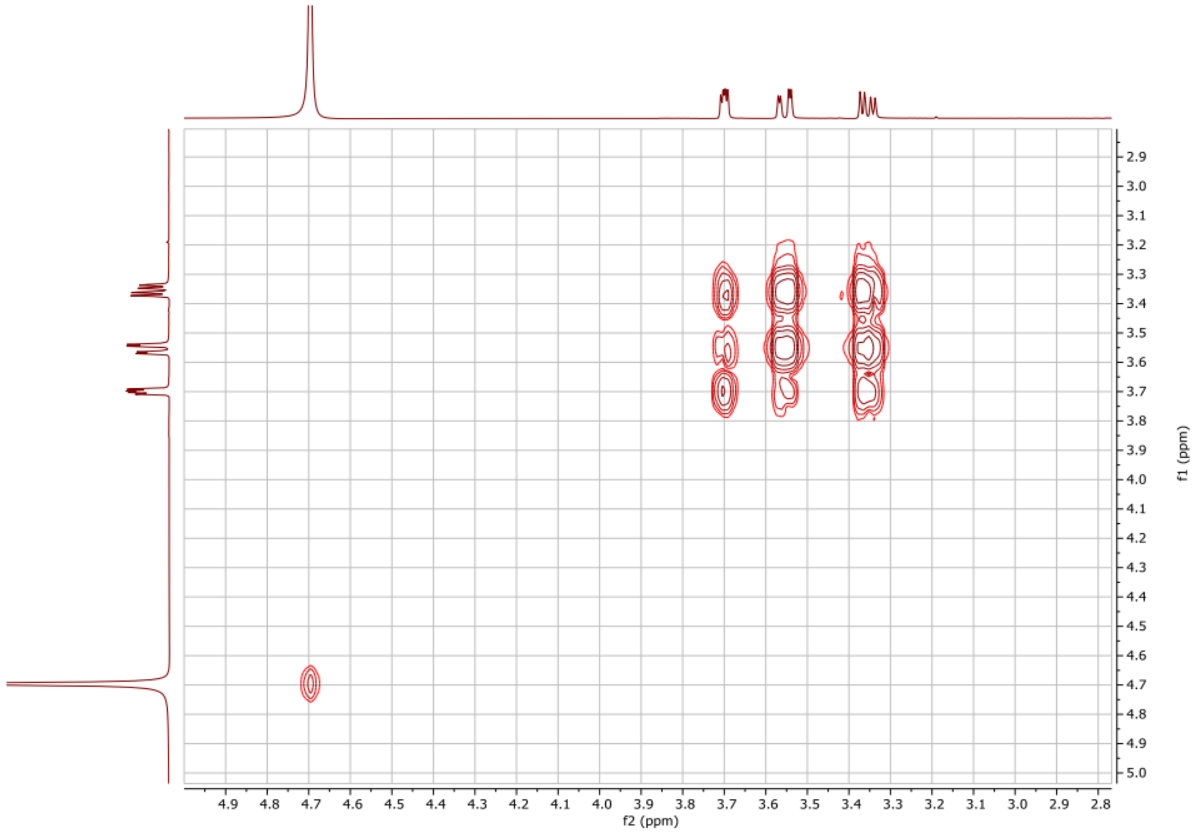

Figure S5. COSY ^{1}H-^{1}H spectrum of compound **1**

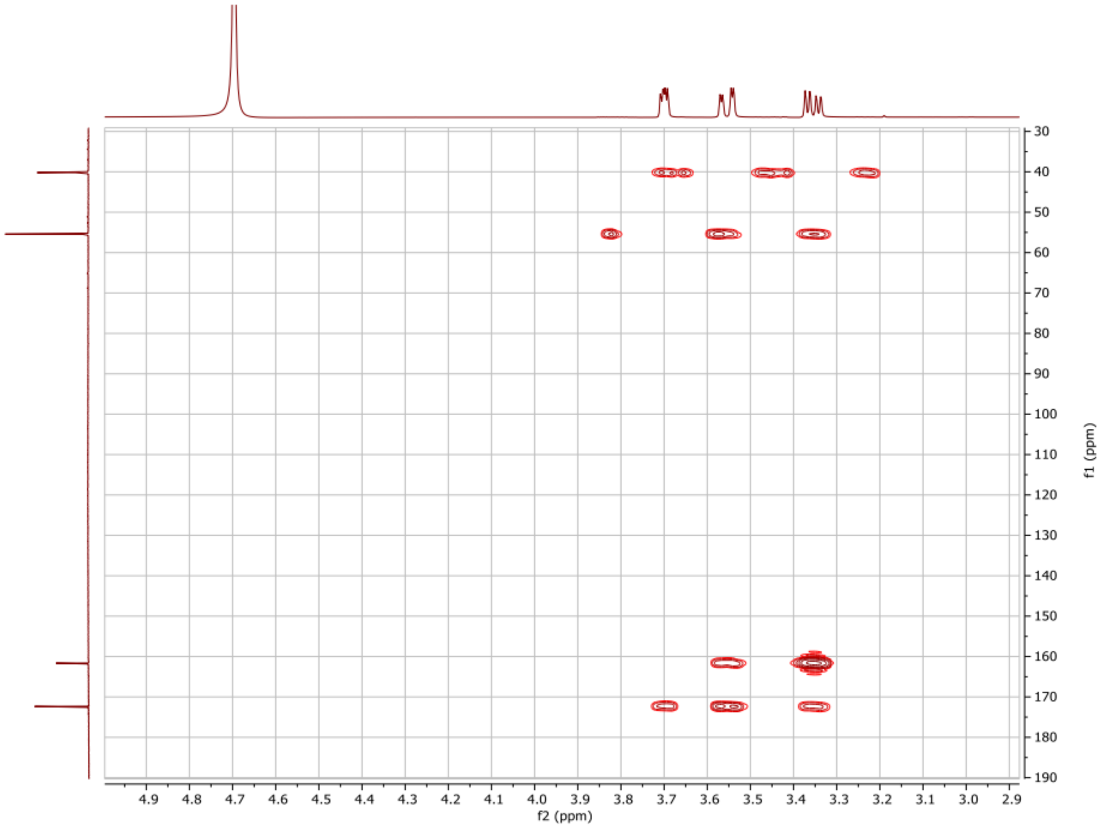

Figure S6. HMBC spectrum of compound **1**

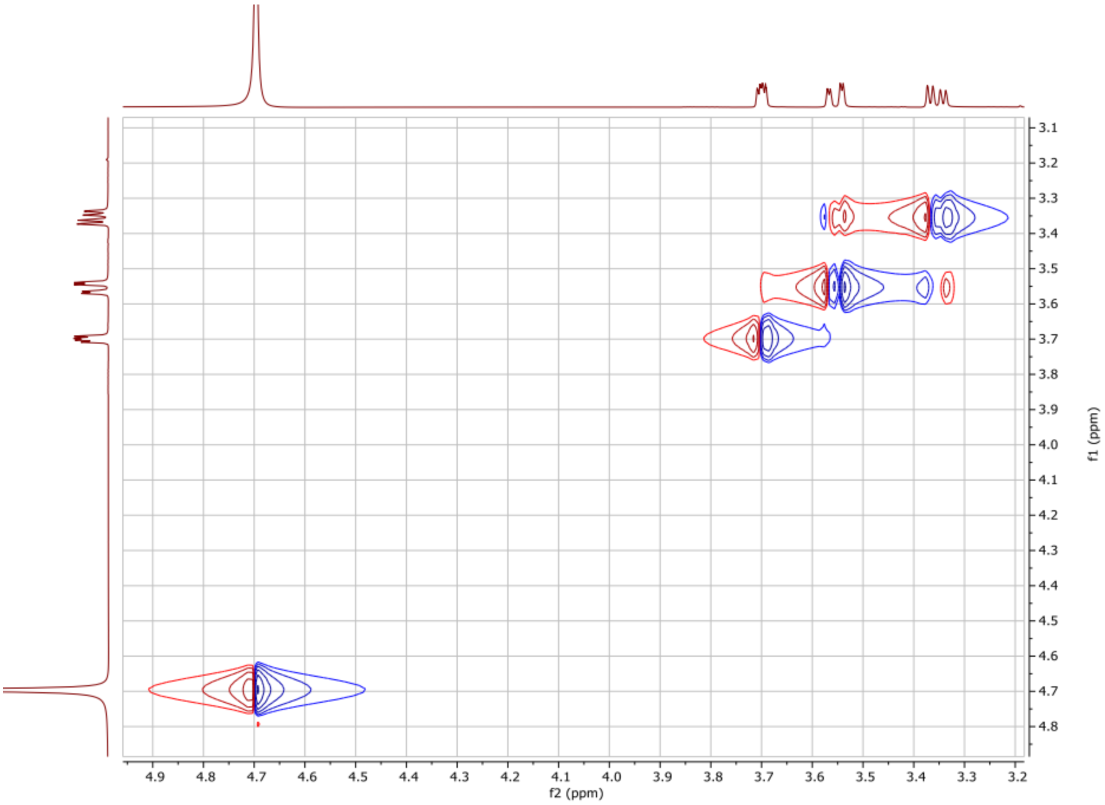

Figure S7. NOESY spectrum of compound **1**

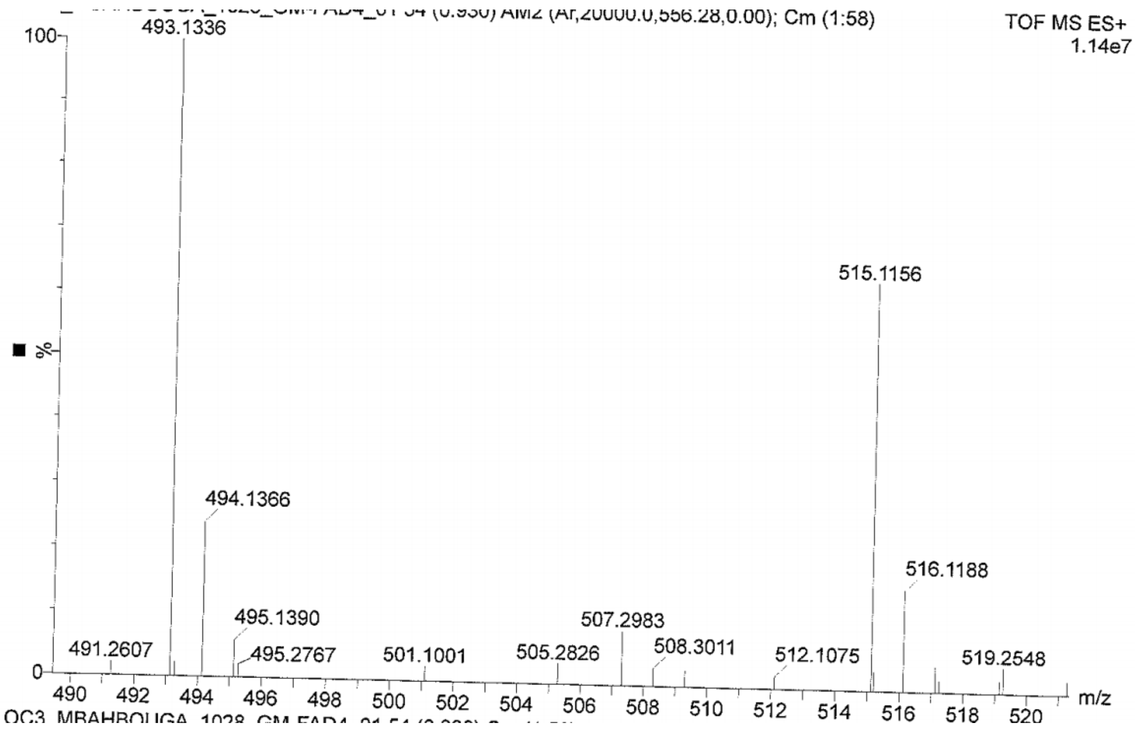

Figure S8. HR-ESI-MS (+) spectrum of compound **2**

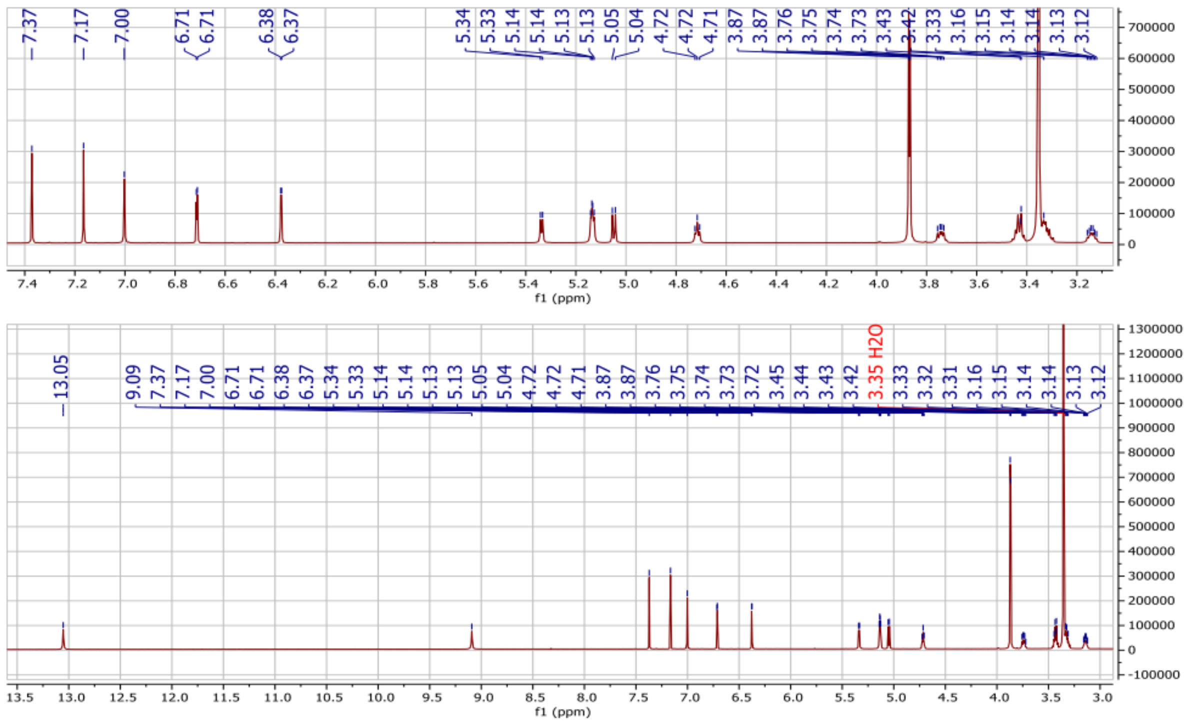

Figure S9. ^{1}H-NMR spectra (600 MHz, DMSO-d_6) of compound **2**

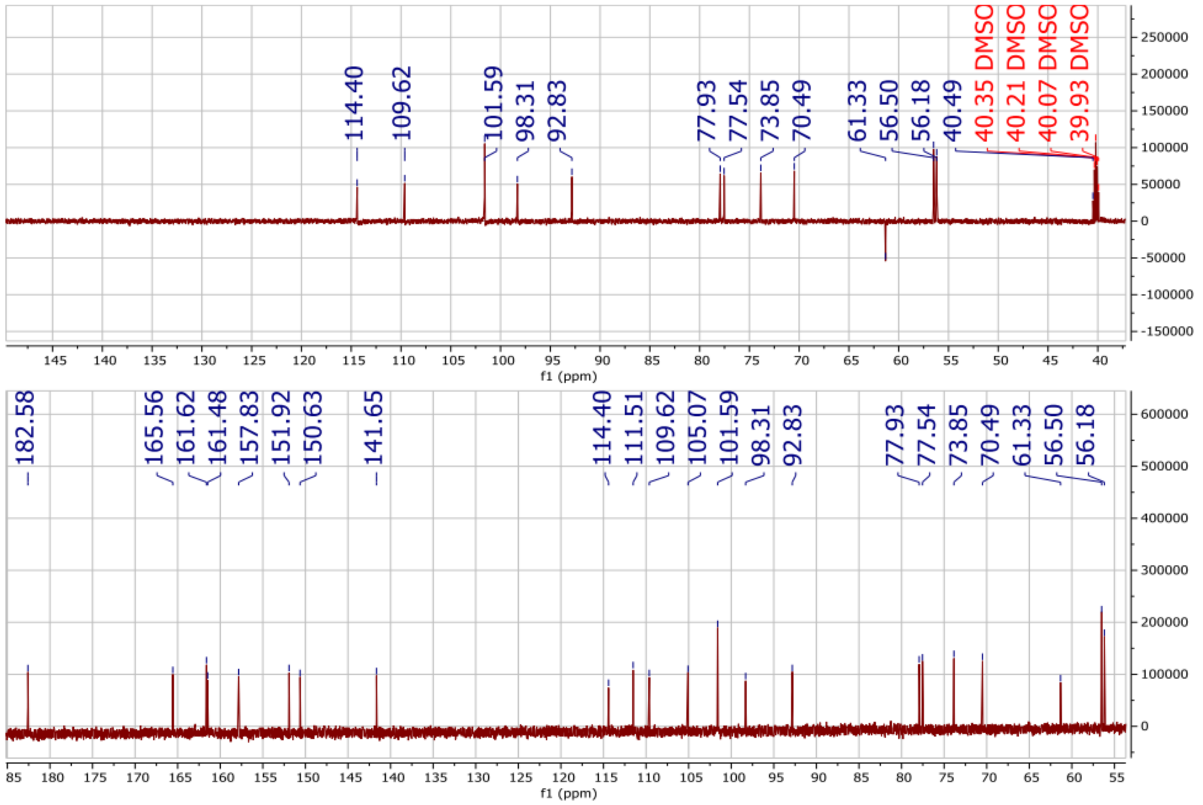

Figure S10. ^{13}C-NMR and DEPT 135 spectra (150 MHz, DMSO- d_6) of compound **2**

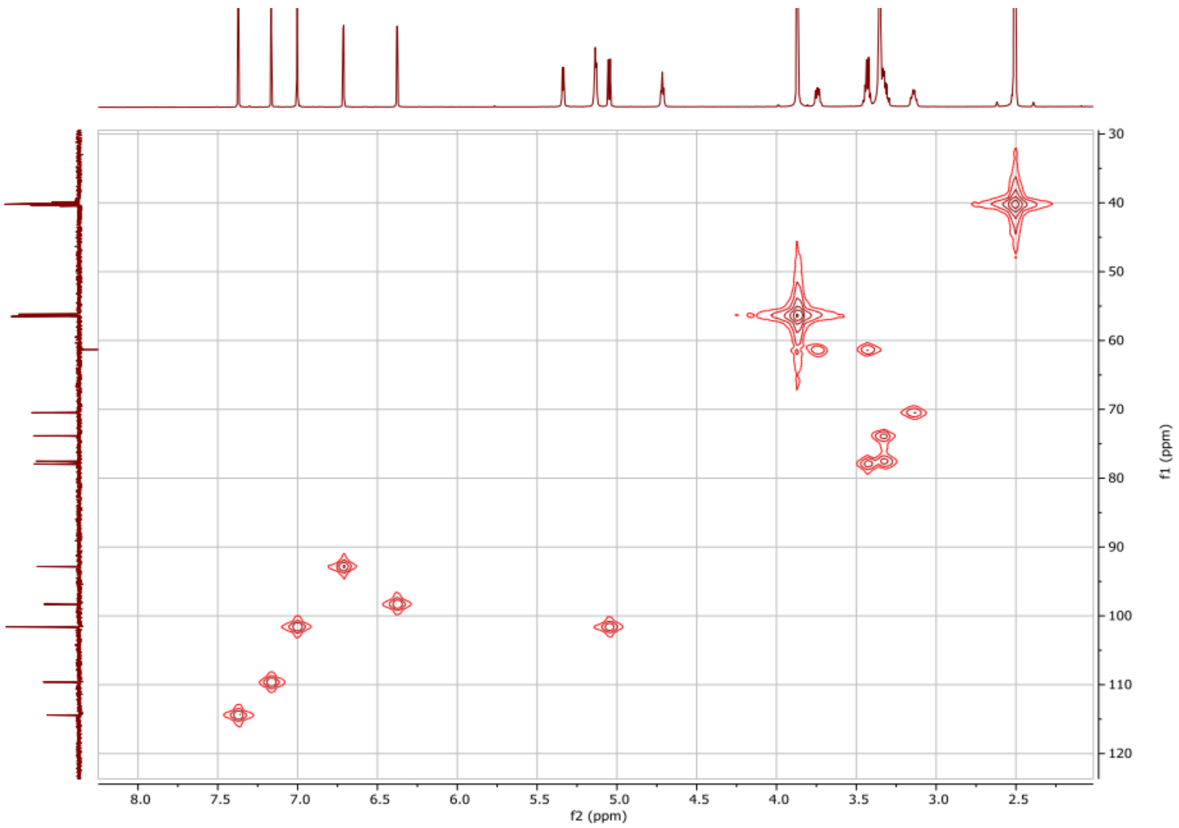

Figure S11. HMQC spectrum of compound **2**

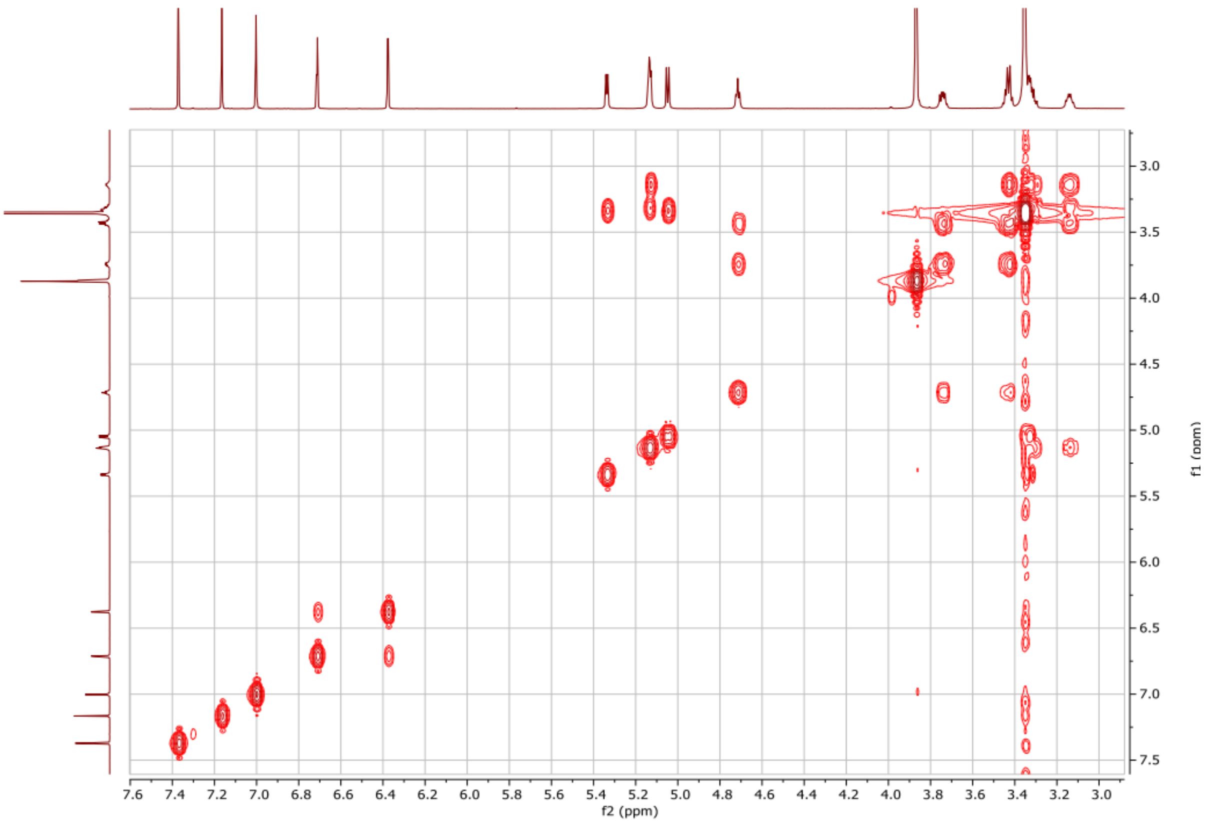

Figure S12. COSY ^{1}H-^{1}H spectrum of compound **2**

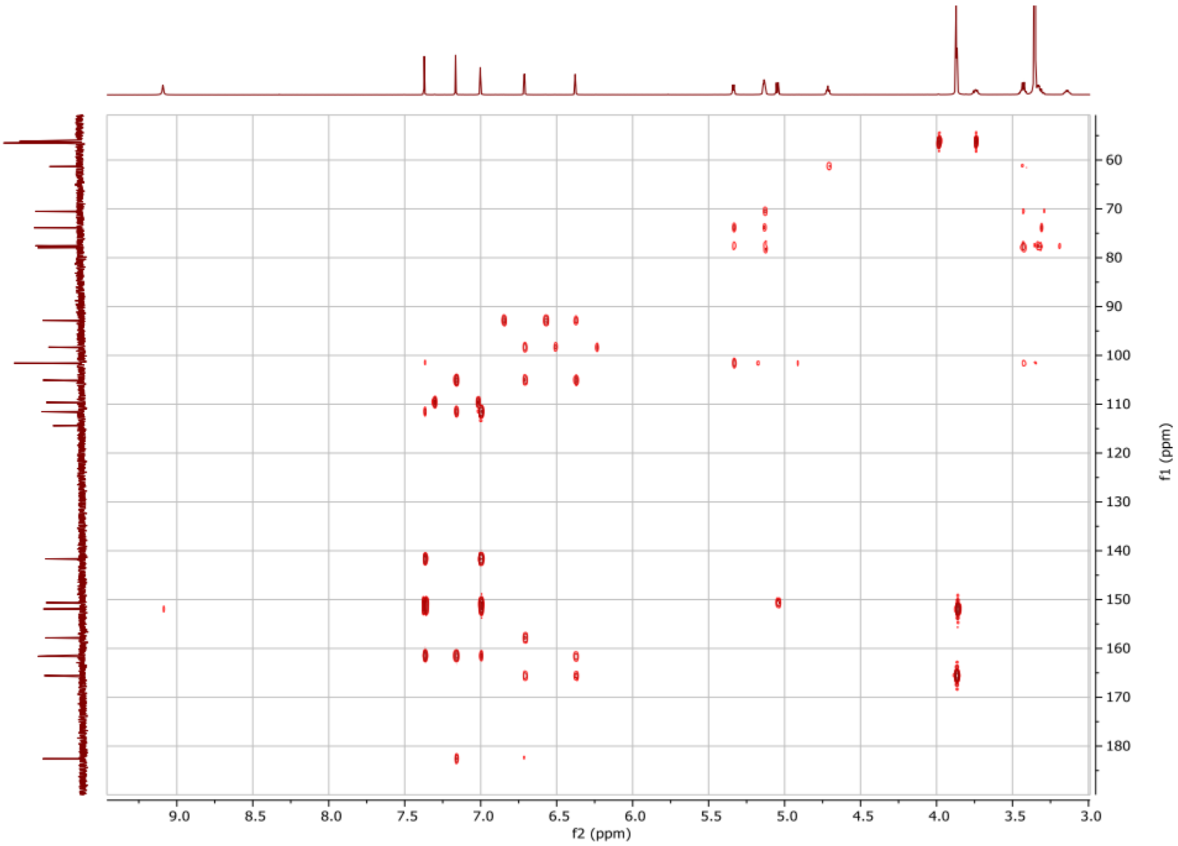

Figure S13. HMBC spectrum of compound **2**

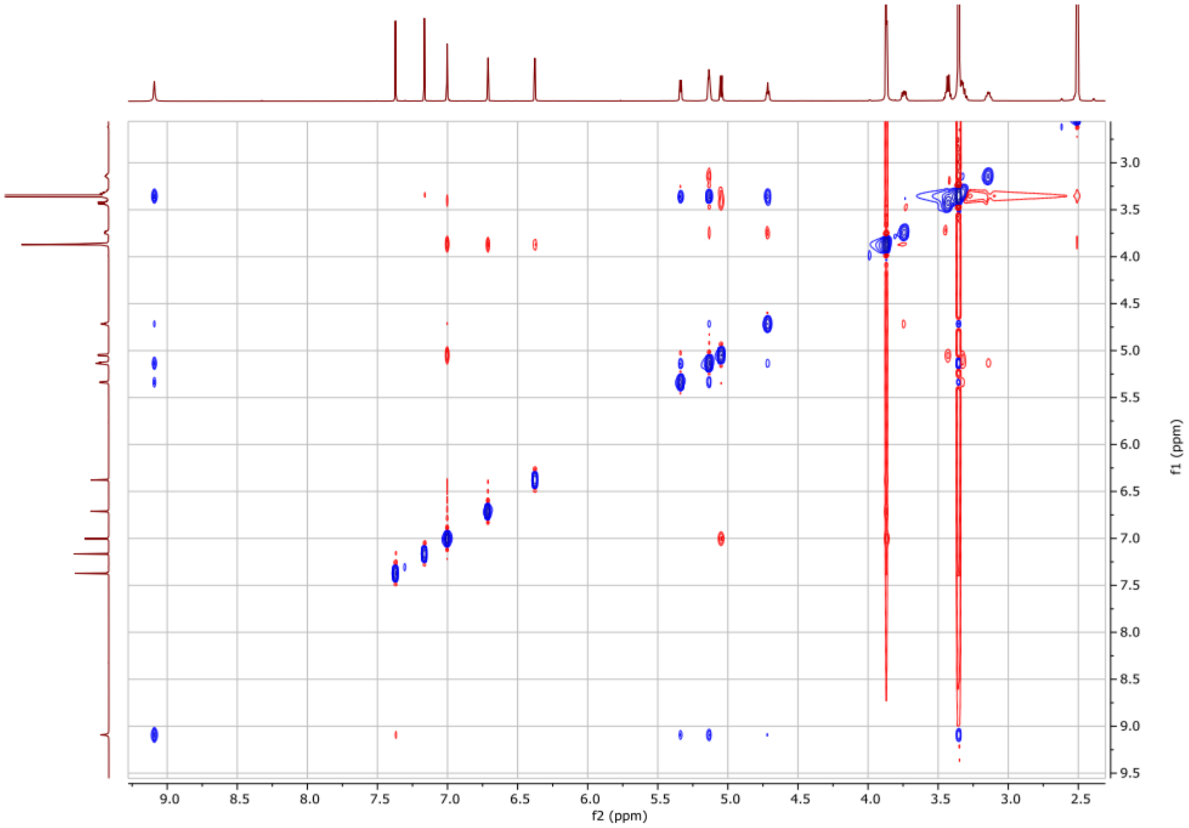

Figure S14. NOESY spectrum of compound **2**

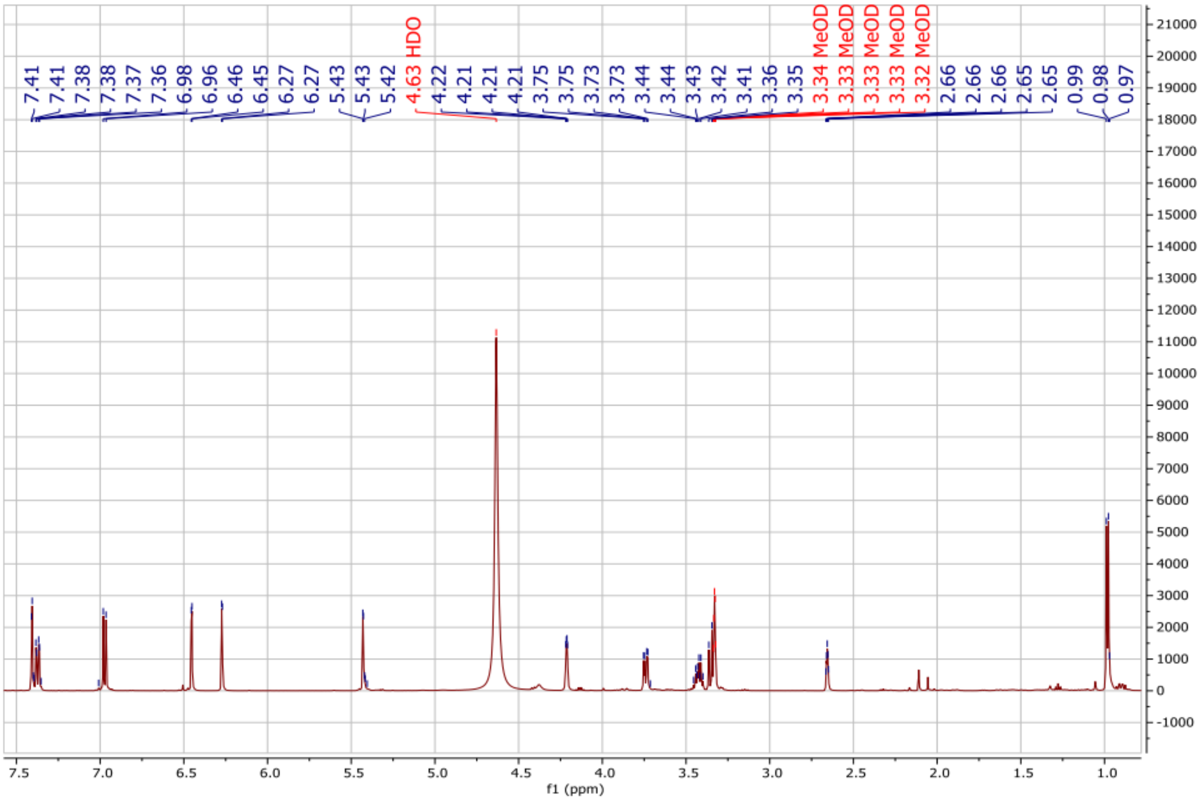

Figure S15. ^{1}H-NMR spectrum (500 MHz, MeOD) of compound **3**

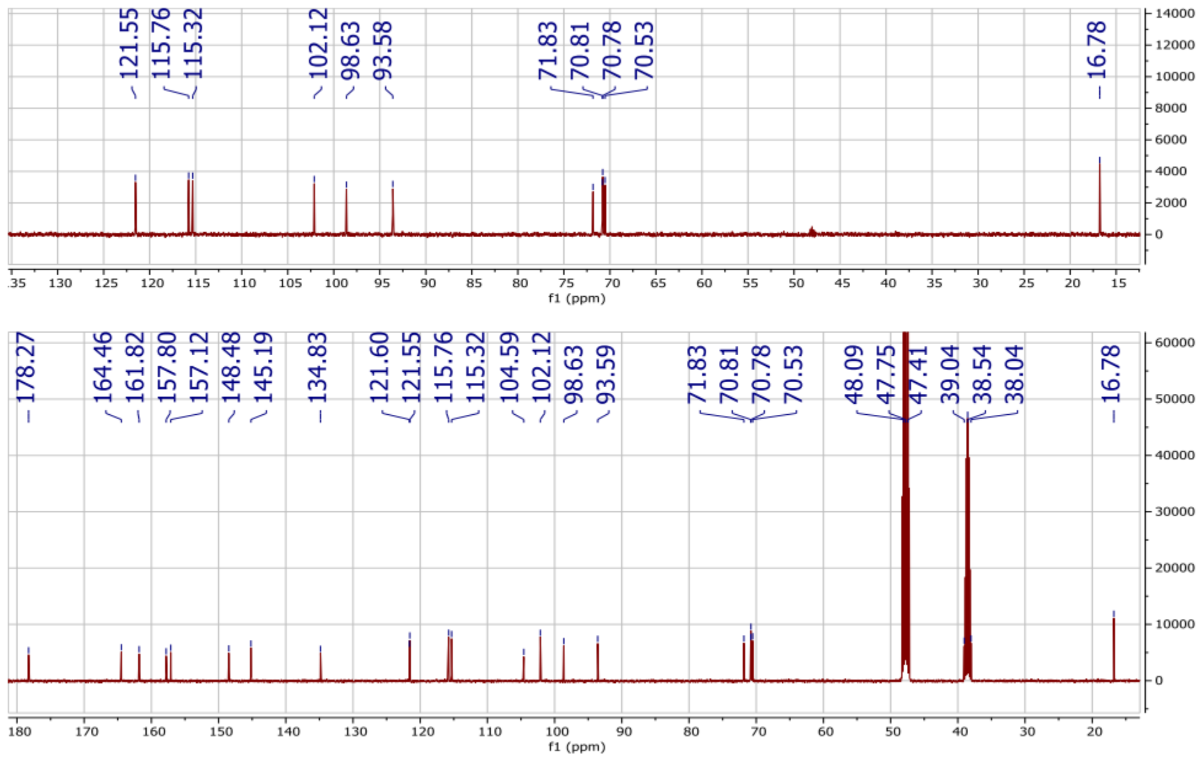

Figure S16. ^{13}C-NMR and DEPT 135 spectra (125 MHz, MeOD) of compound **3**

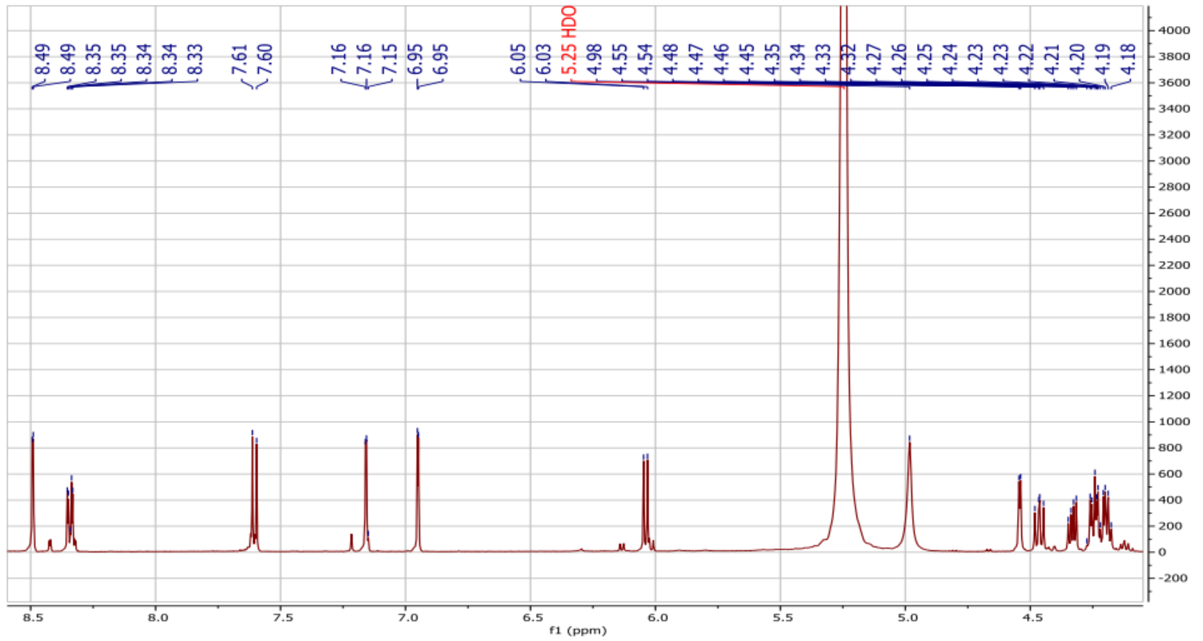

Figure S17. ^{1}H-NMR spectrum (500 MHz, MeOD) of compound **4**

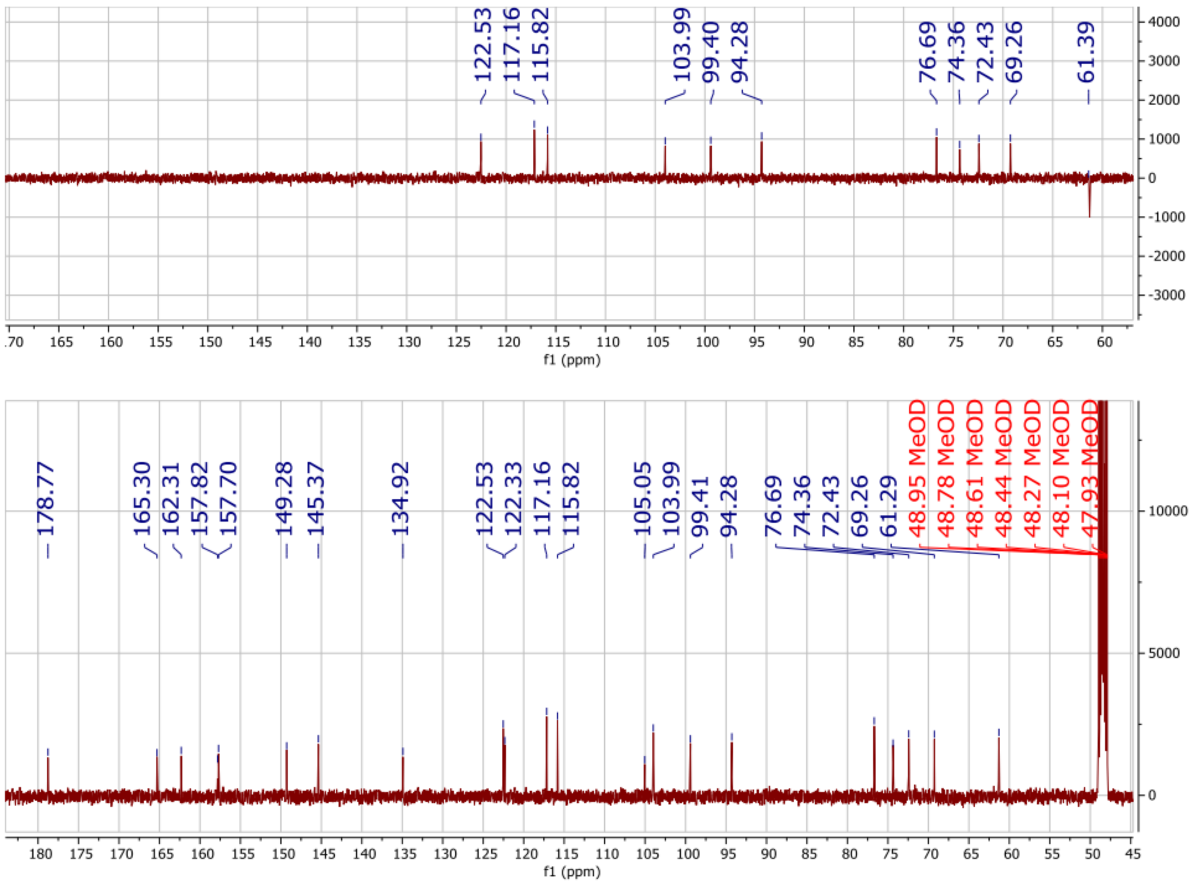

Figure S18. ^{13}C-NMR and DEPT135 spectra (125 MHz, MeOD) of compound **4**

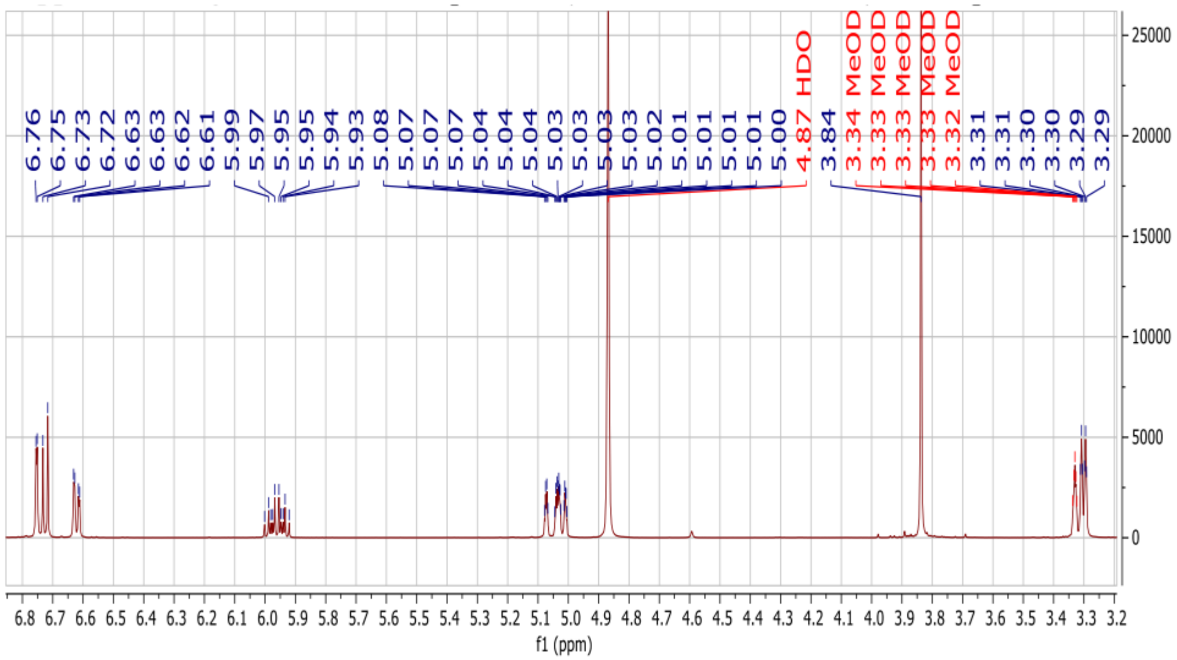

Figure S19. ^{1}H-NMR spectrum (MeOD, 500 MHz) of compound **5**

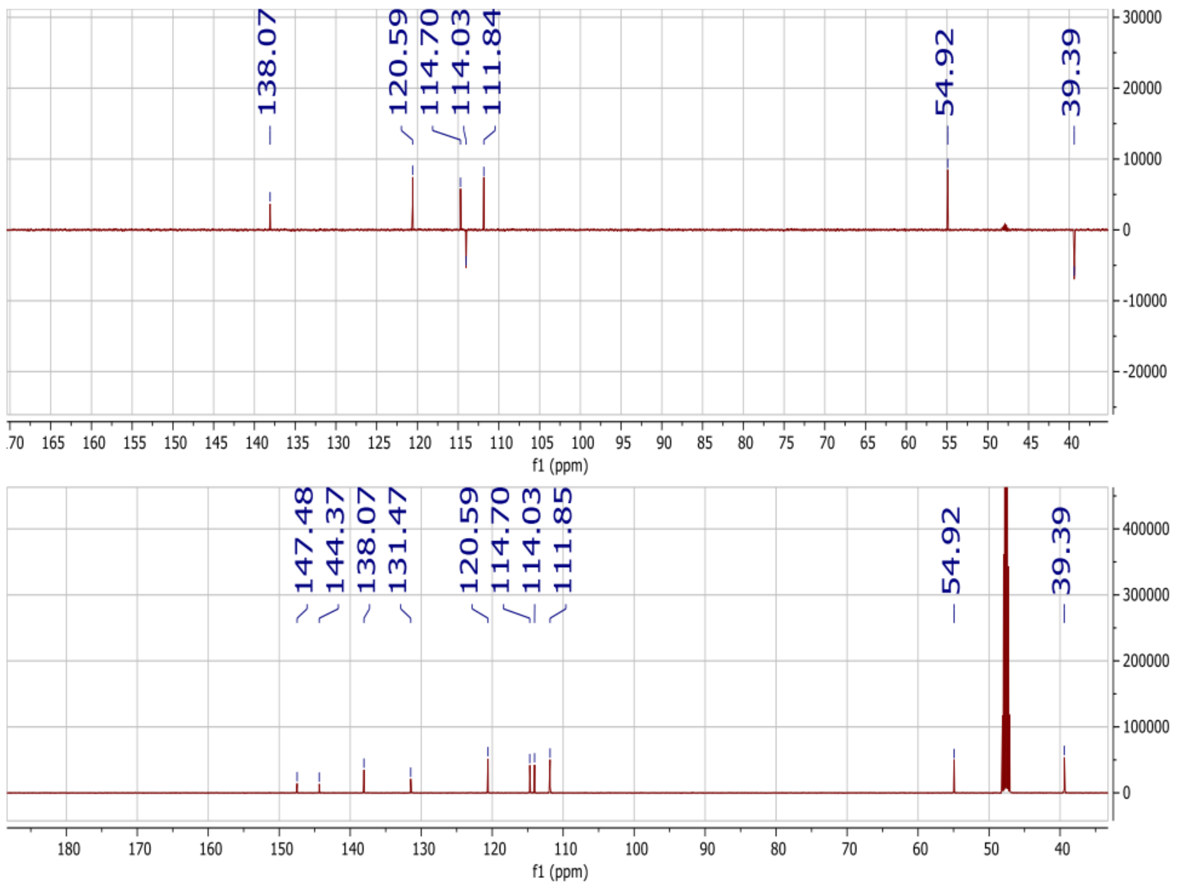

Figure S20. ^{13}C-NMR and DEPT 135 spectra (MeOD, 125 MHz) of compound **5**

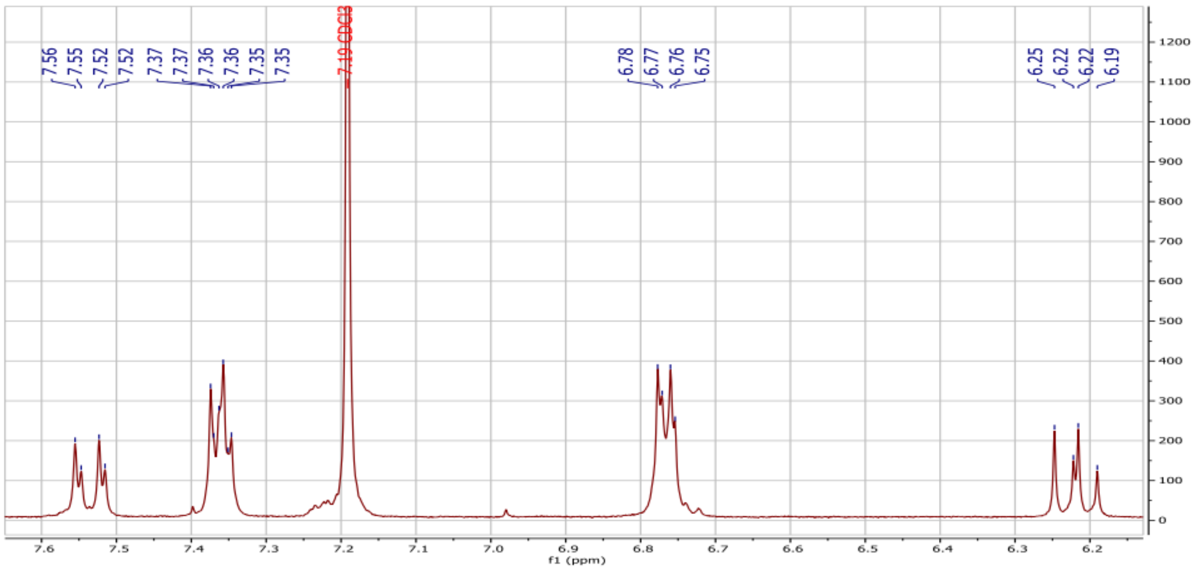

Figure S21. ^{1}H-NMR spectrum ($CDCl_3$; 500 MHz) of compounds **6** and **7**

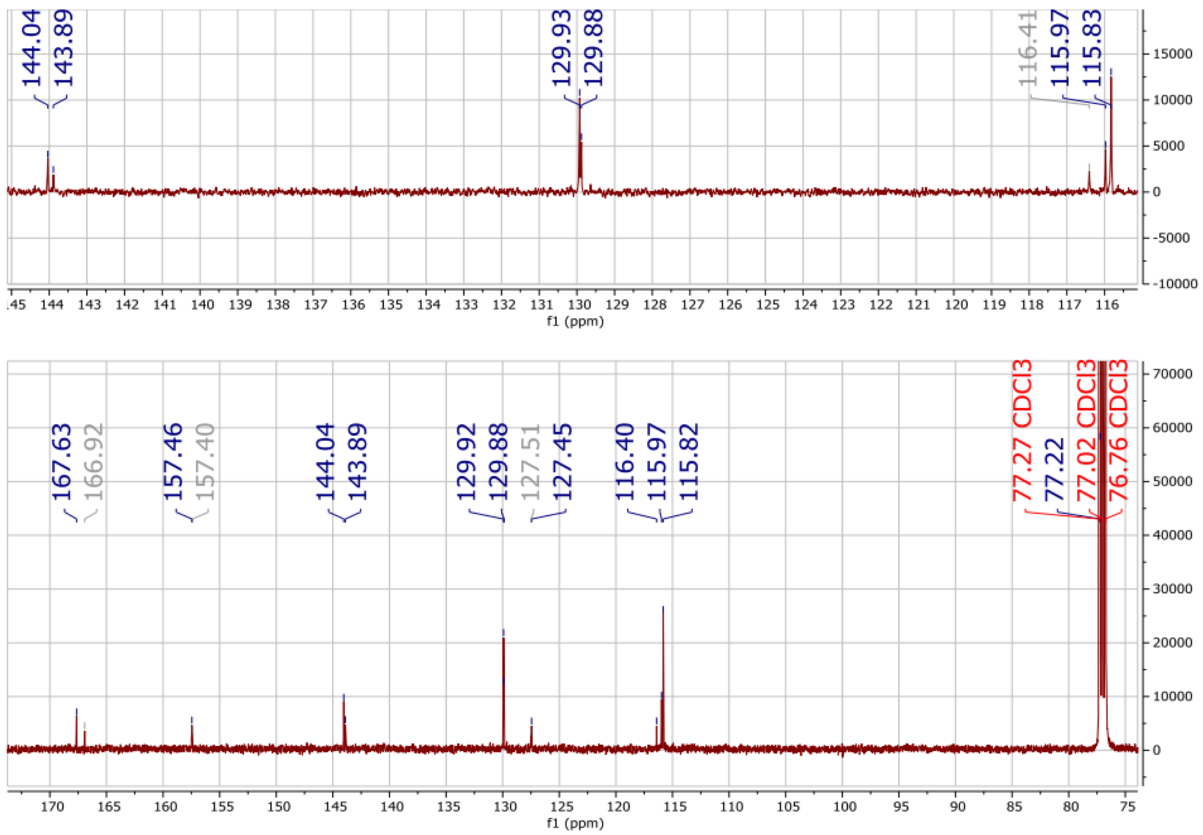

Figure S22. ^{13}C-NMR and DEPT 135 spectra ($CDCl_3$; 125 MHz) of compounds **6** and **7**

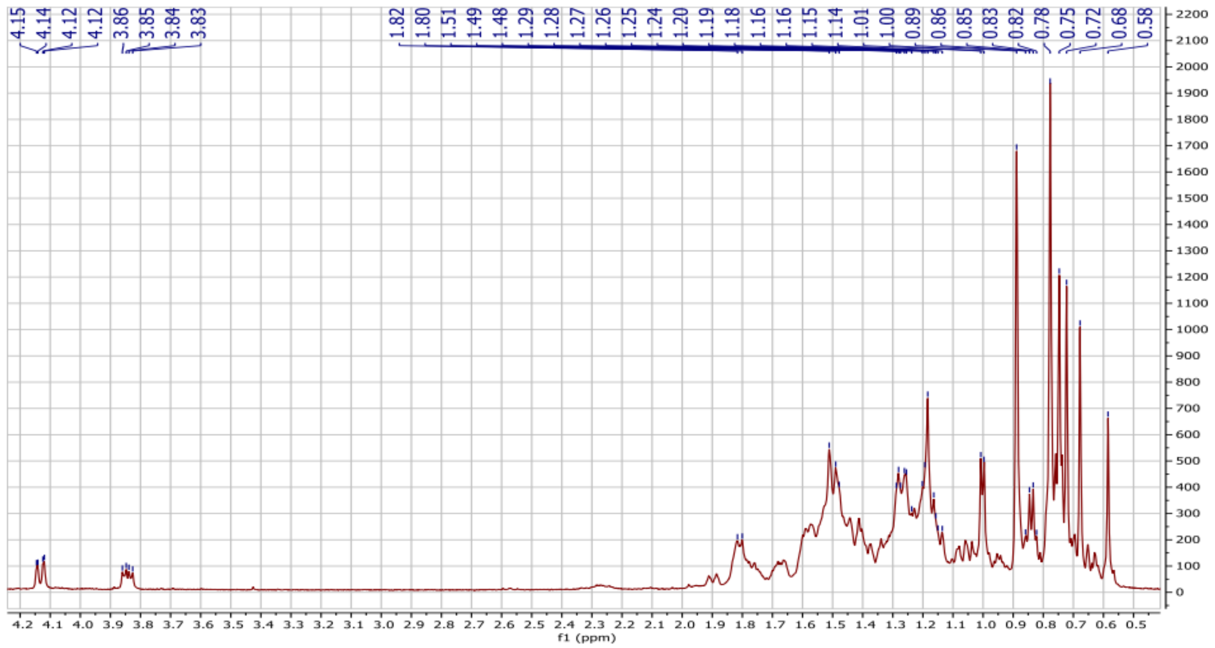

Figure S23. ^{1}H-NMR spectrum ($CDCl_3$, 500 MHz) of compound **8**

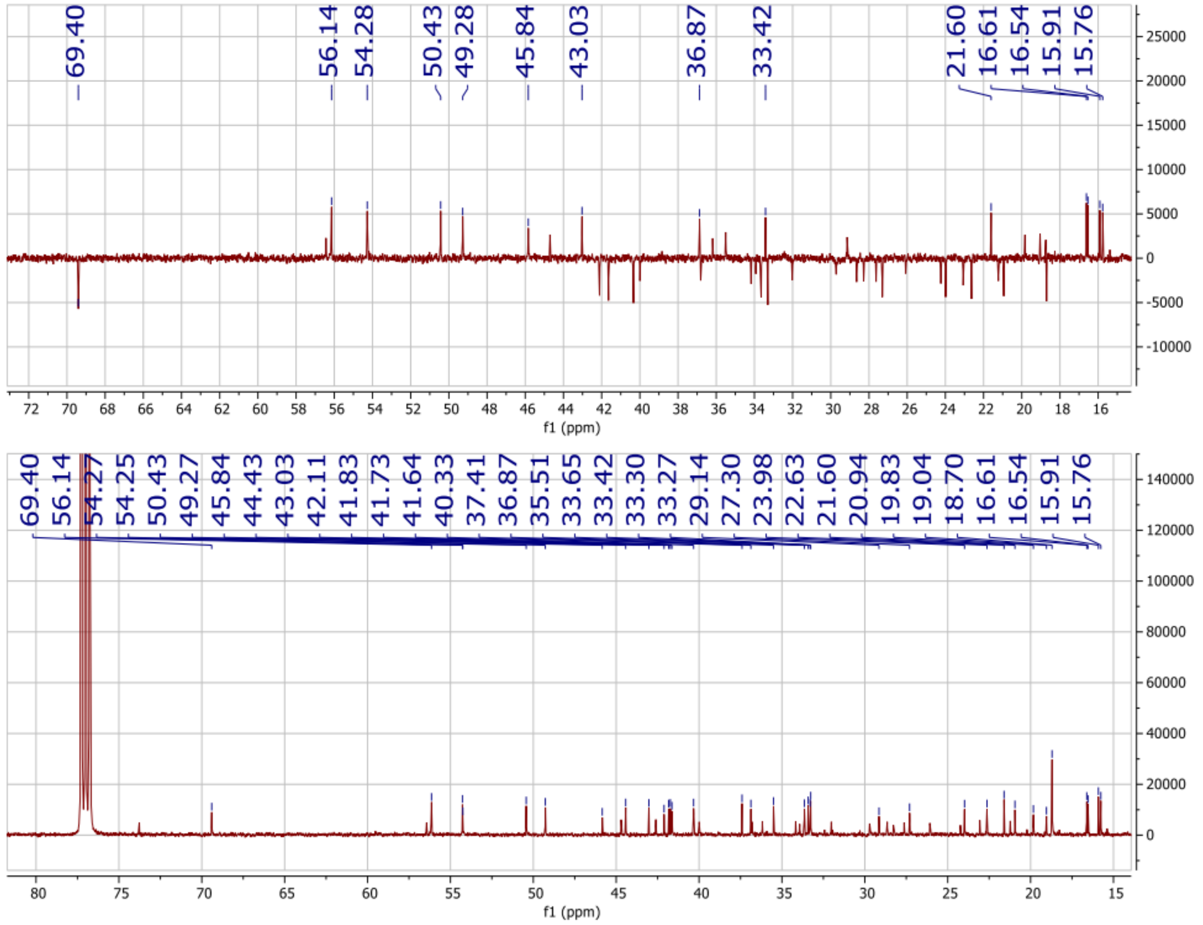

Figure S24. ^{13}C-NMR &DEPT 135 spectra ($CDCl_3$, 125 MHz) of compound **8**

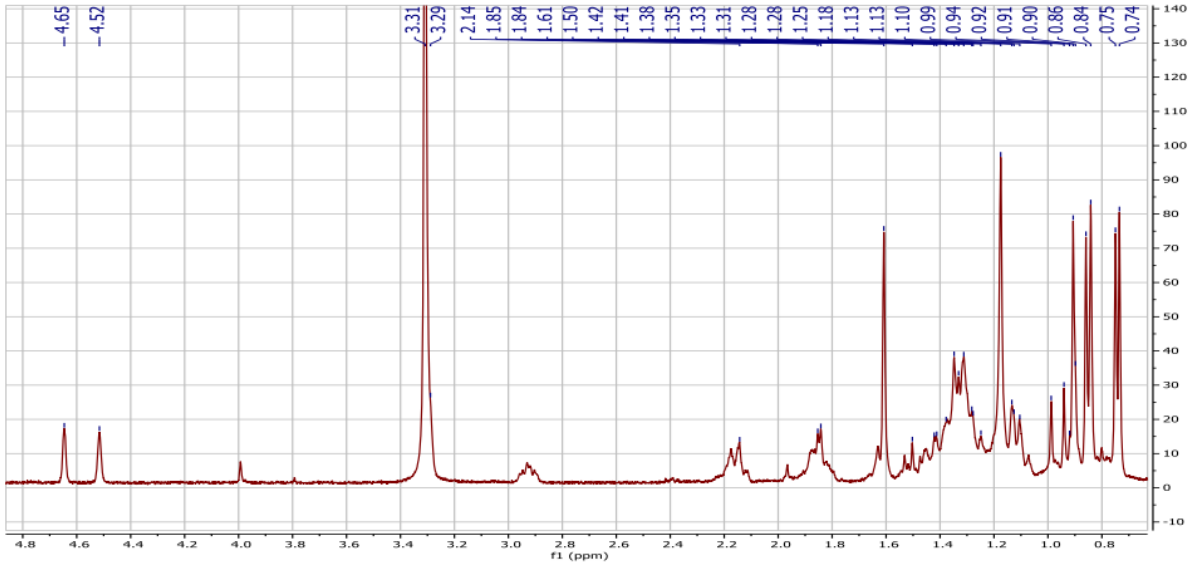

Figure S25. ^{1}H-NMR spectrum ($CDCl_3$, 400 MHz) of compound **9**

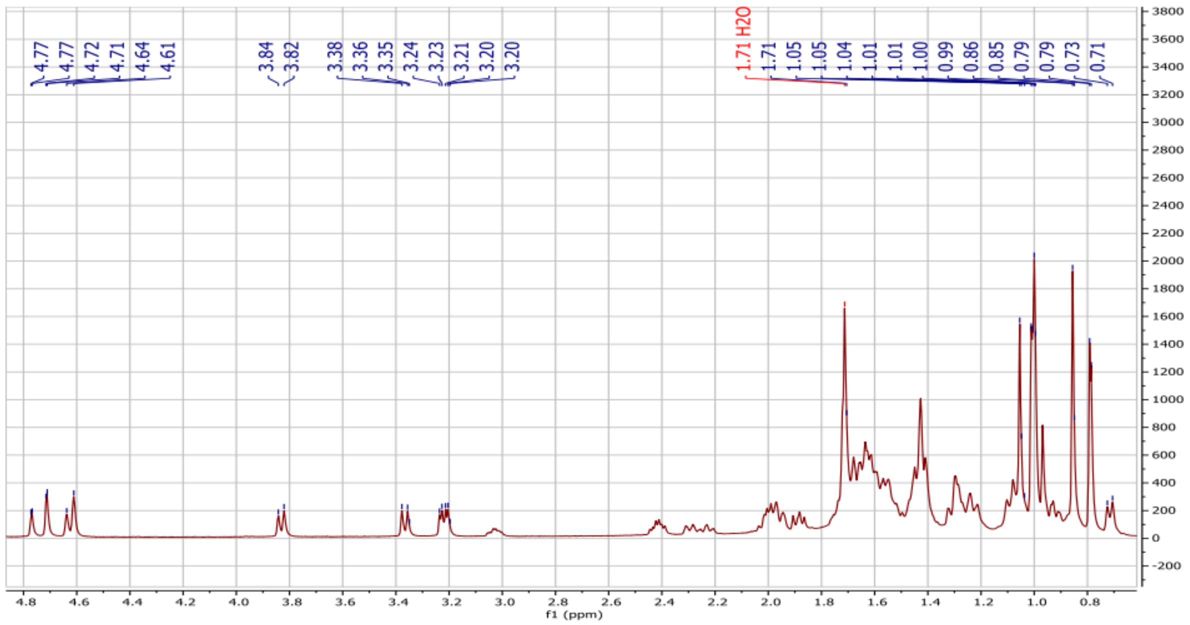

Figure S26. ^{1}H-NMR spectrum ($CDCl_3$, 500 MHz) of compound **10**

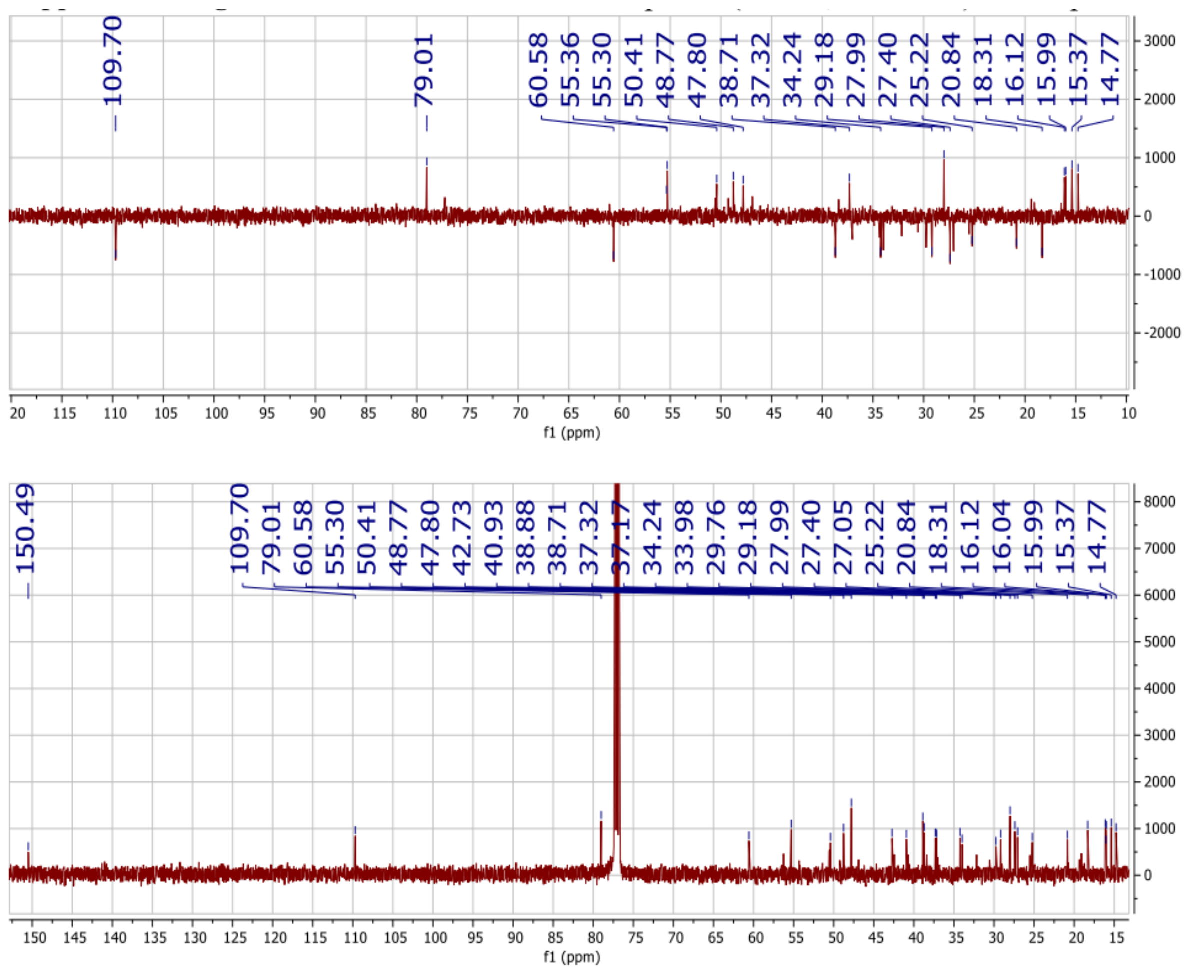

Figure S27. ^{13}C-NMR spectrum ($CDCl_3$, 125 MHz) of compound **10**

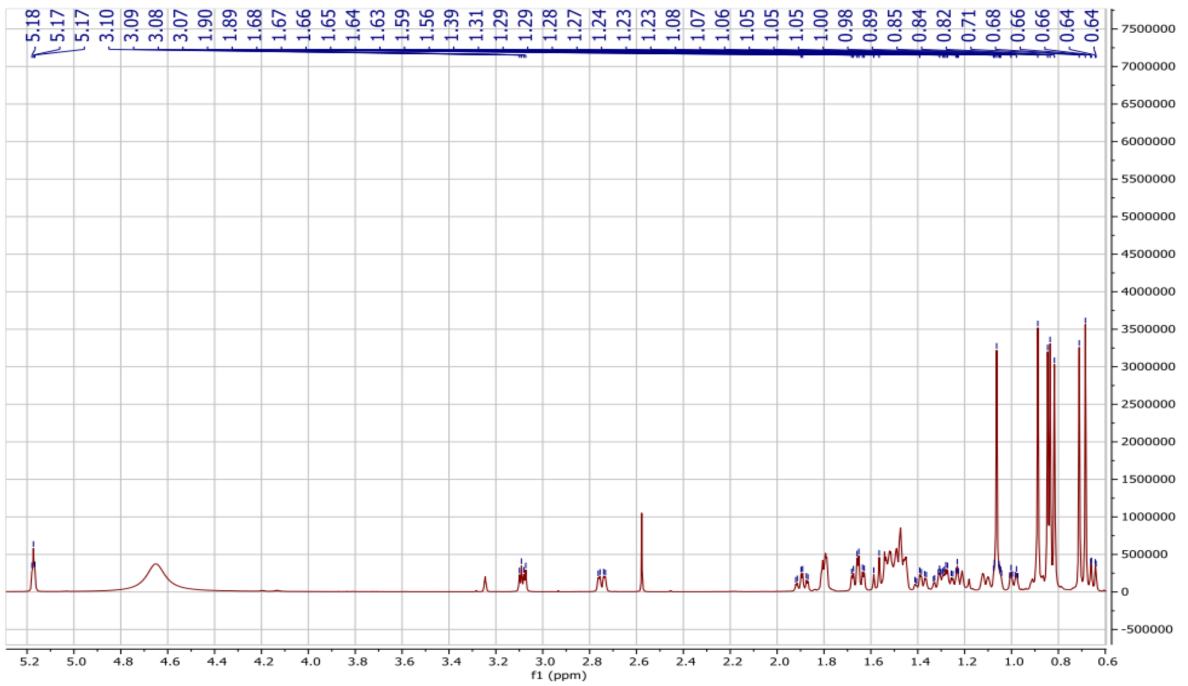

Figure S28. ^{1}H-NMR spectrum (MeOD; 600 MHz) of compound **11**

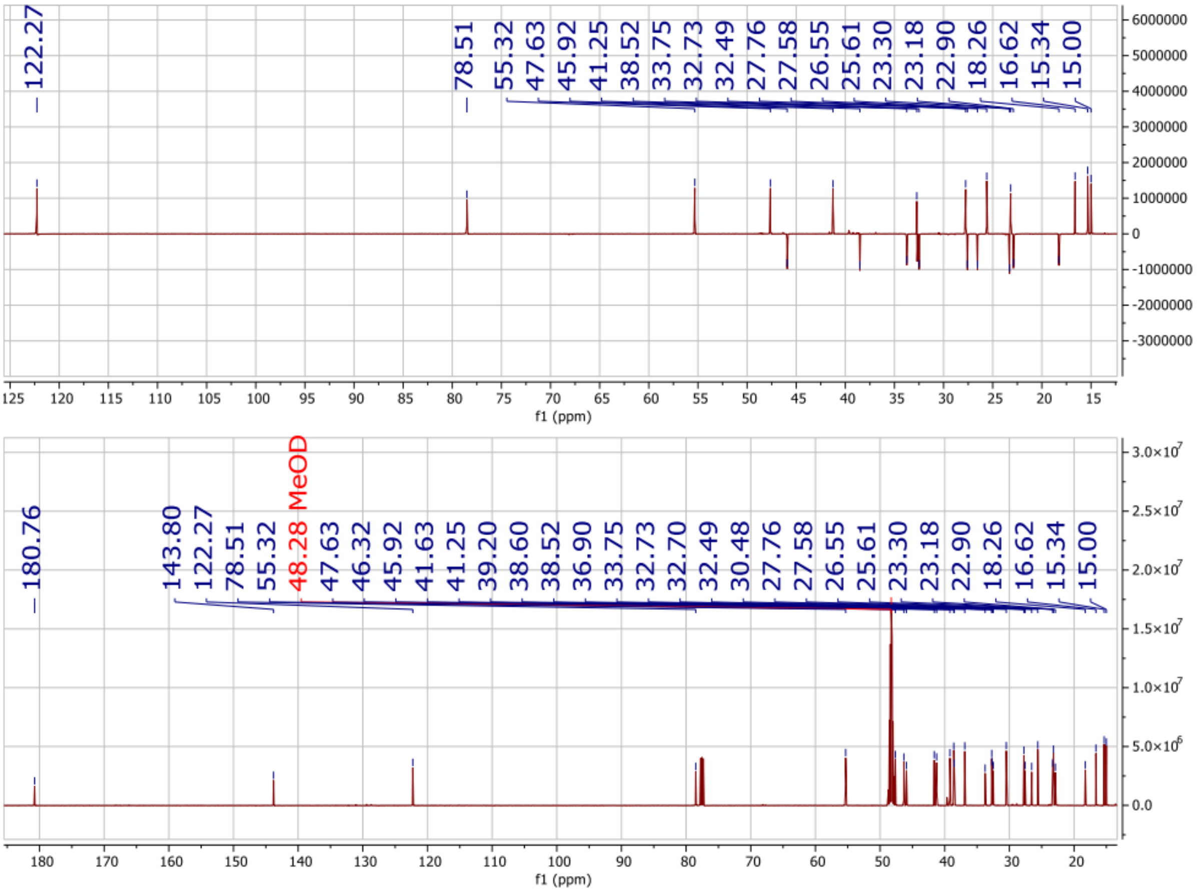

Figure S29. ^{13}C-NMR and DEPT 135 spectra (MeOD; 150 MHz) of compound **11**

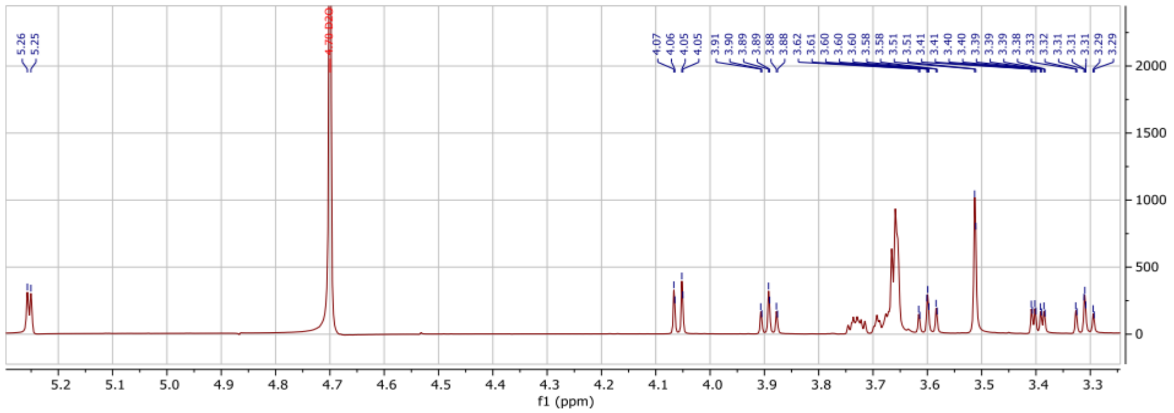

Figure S30. ^{1}H-NMR spectrum (D_2O, 600 MHz) of compound **12**

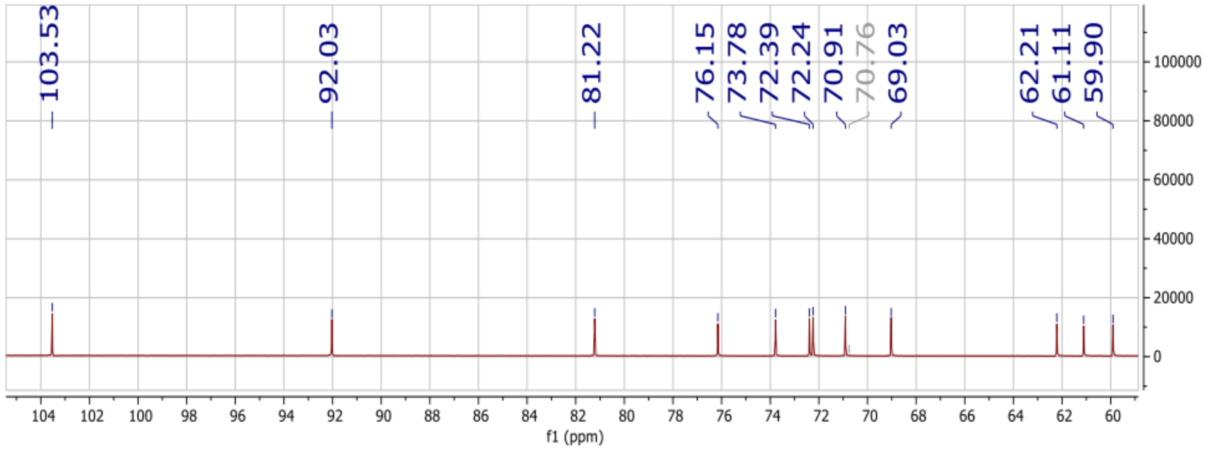

Figure S31. ^{13}C-NMR spectrum (D_2O, 150 MHz) of compound **12**

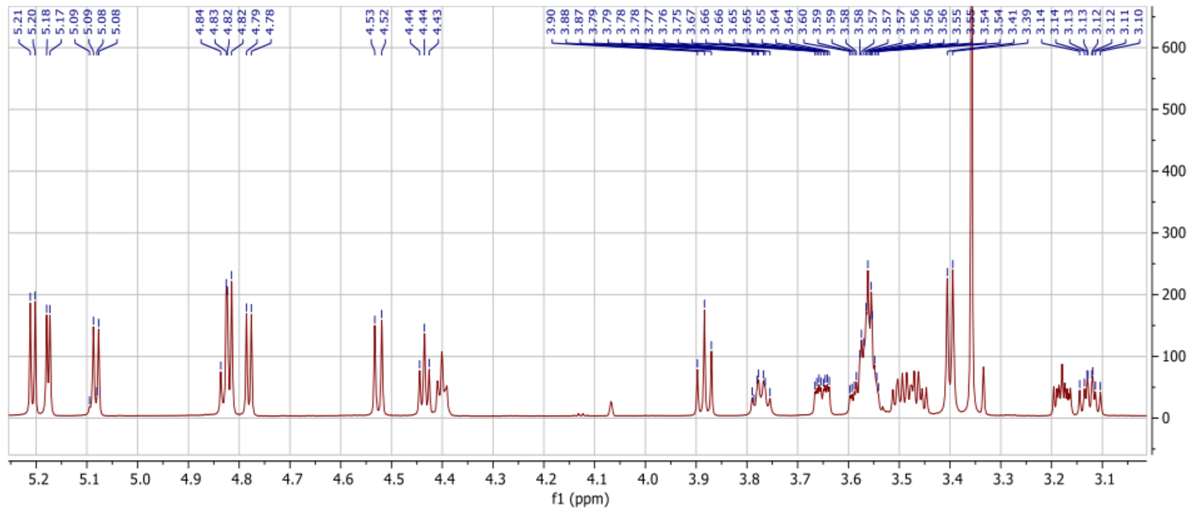

Figure S32. ^{1}H-NMR spectrum (DMSO-d_6, 600 MHz) of compound **13**

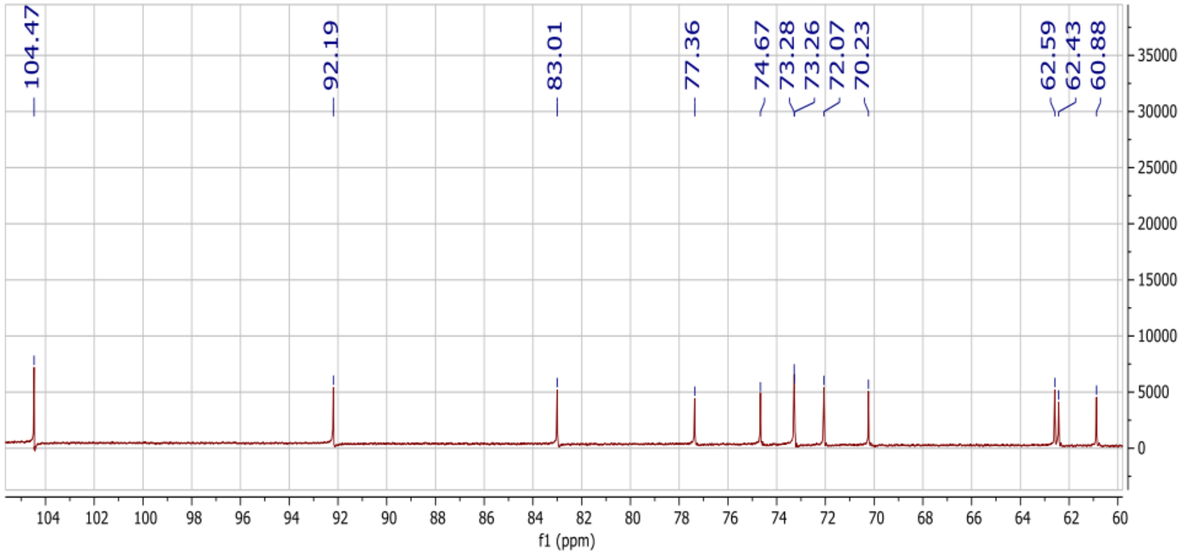

Figure S33. ^{13}C-NMR spectrum (DMSO-d_6, 150 MHz) of compound **13**

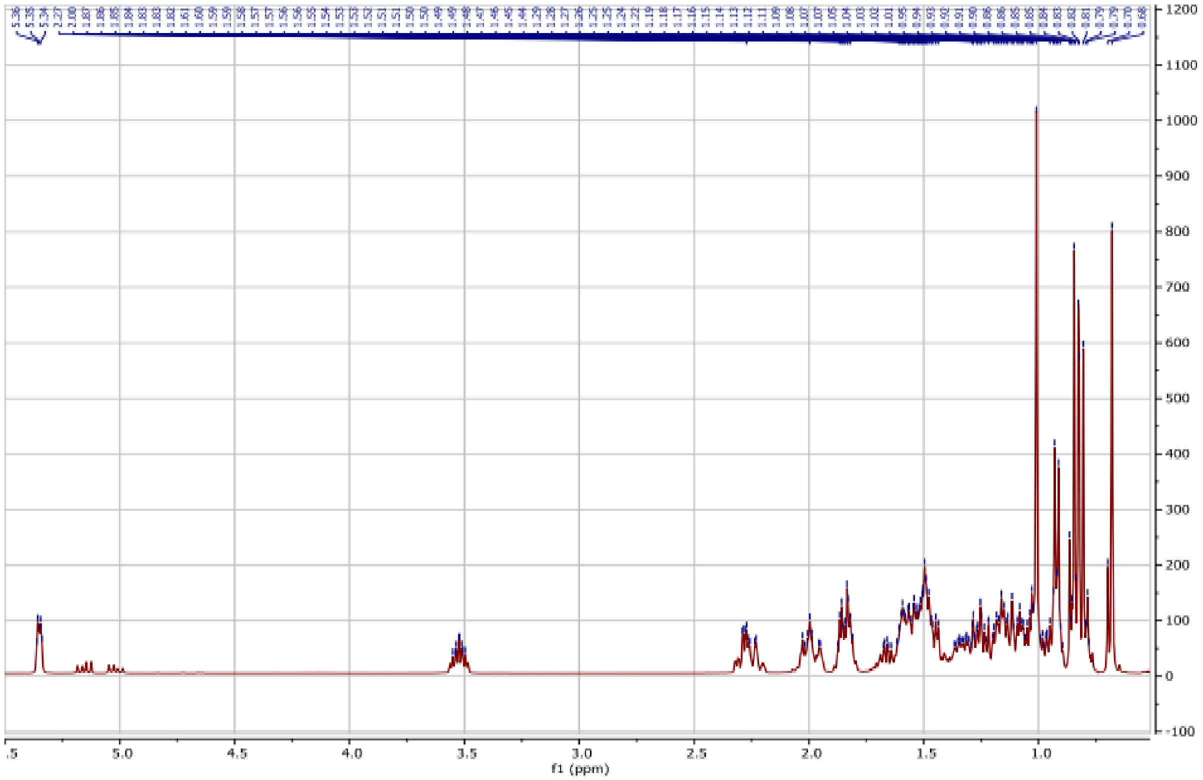

Figure S34. ^{1}H-NMR spectrum ($CDCl_3$, 500 MHz) of compound **14** and **16**

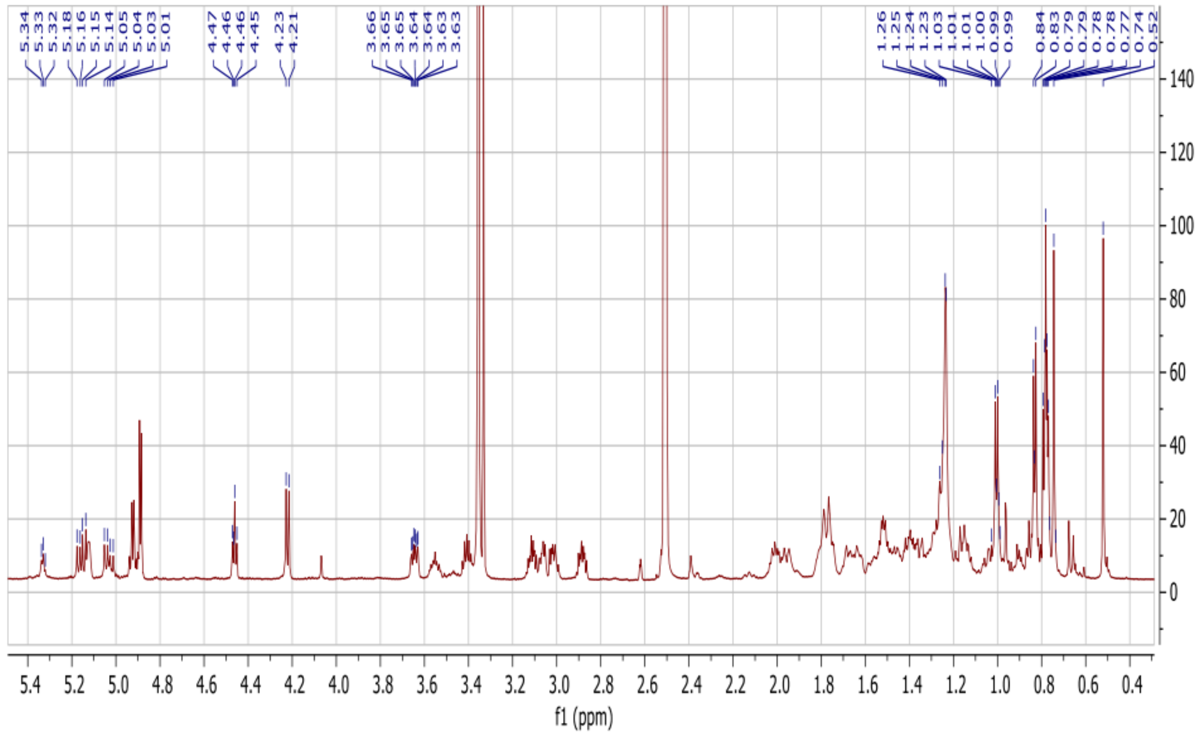

Figure S35. ^{1}H-NMR spectrum (DMSO-d_6, 500 MHz) of compounds **15** and **17**

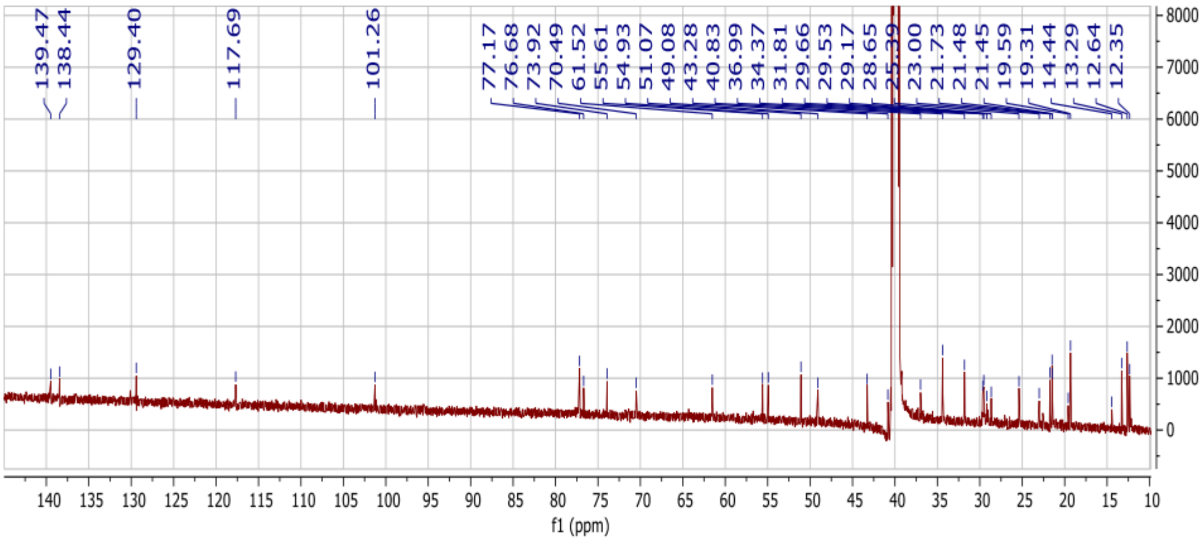

Figure S36. ^{13}C-NMR spectrum (DMSO-d_6, 125 MHz) of compound **17**

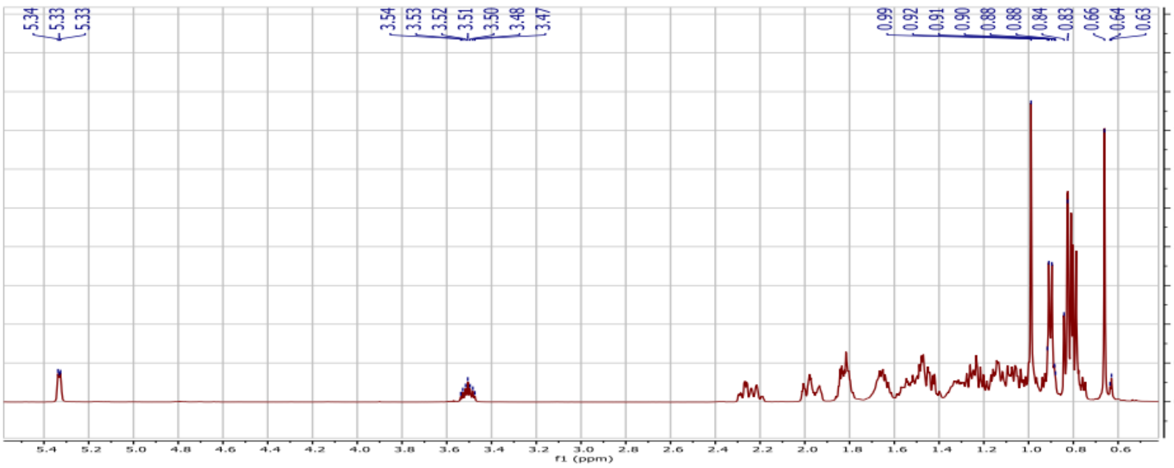

Figure S37. ^{1}H-NMR spectrum ($CDCl_3$, 500 MHz) of compound **18**

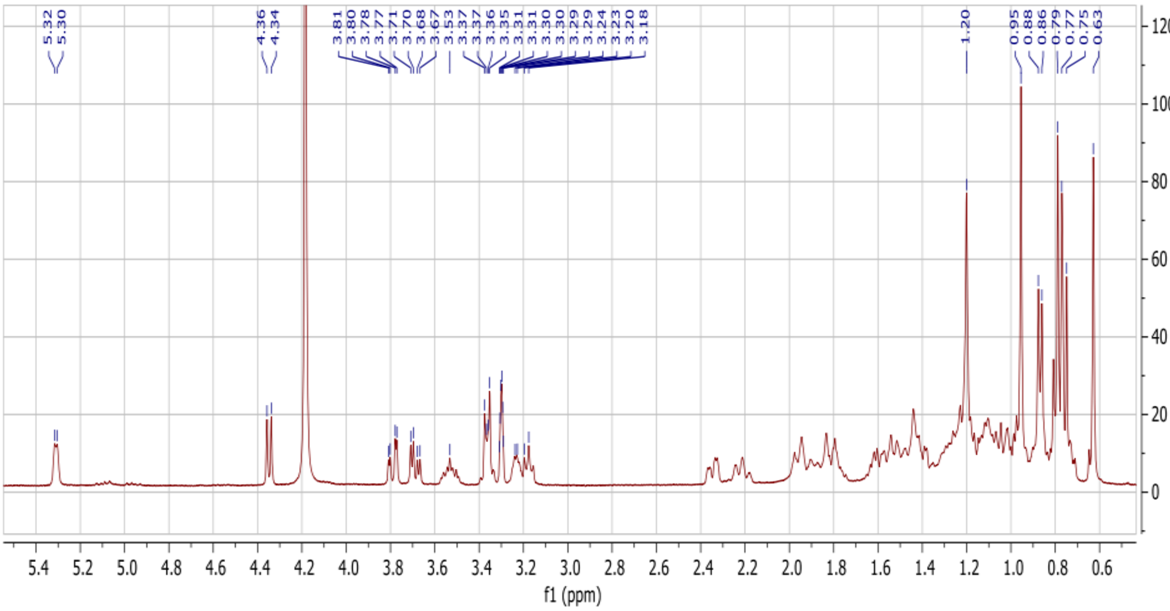

Figure S38. ^{1}H-NMR spectrum (MeOD, 500 MHz) of compound **19**

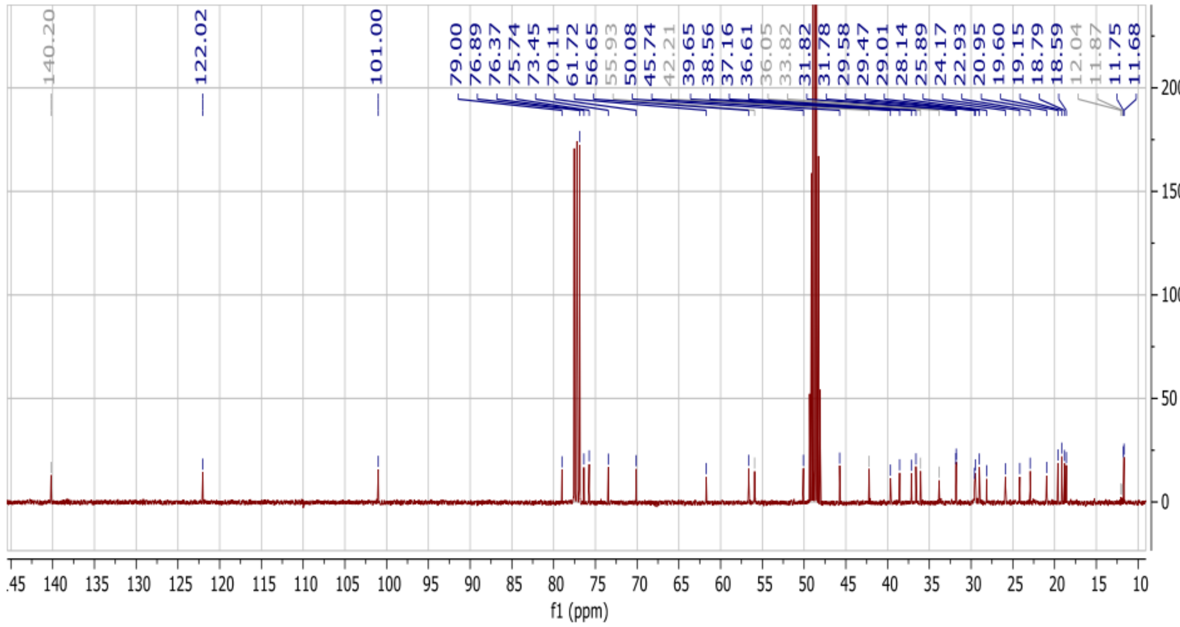

Figure S39. ^{13}C-NMR spectrum ($CDCl_3$/MeOD, 125 MHz) of compound **19**

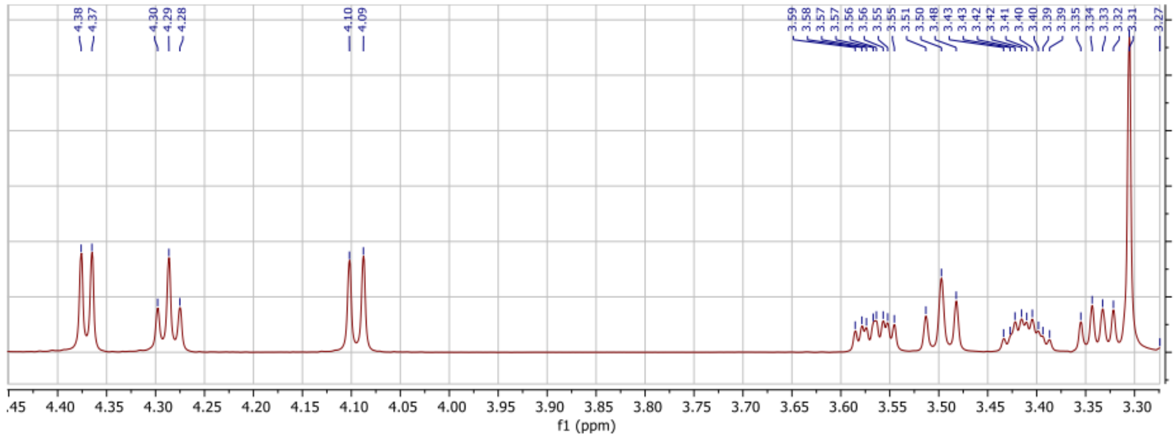

Figure S40. ^{1}H-NMR spectrum (DMSO-d_6, 500 MHz) of compound **20**

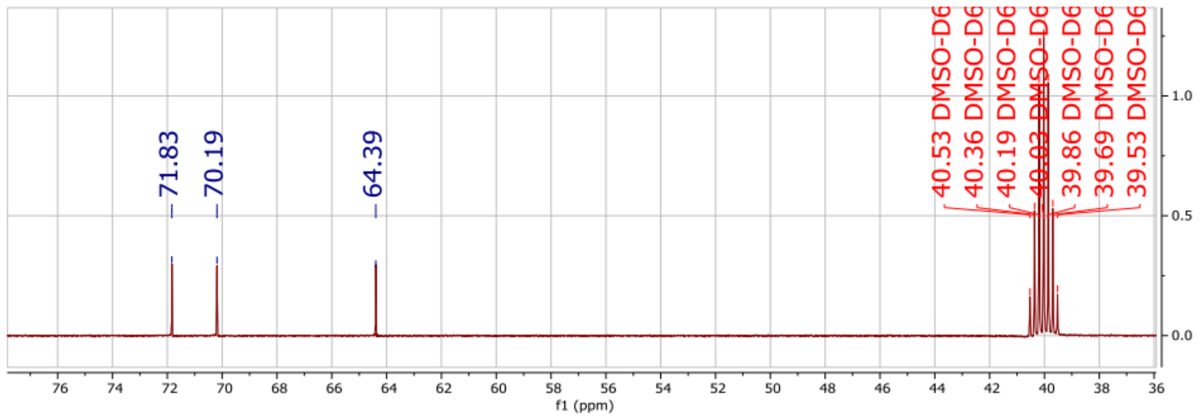

Figure S41: ^{13}C-NMR spectrum (DMSO-d_6, 500 MHz) of compound **20**

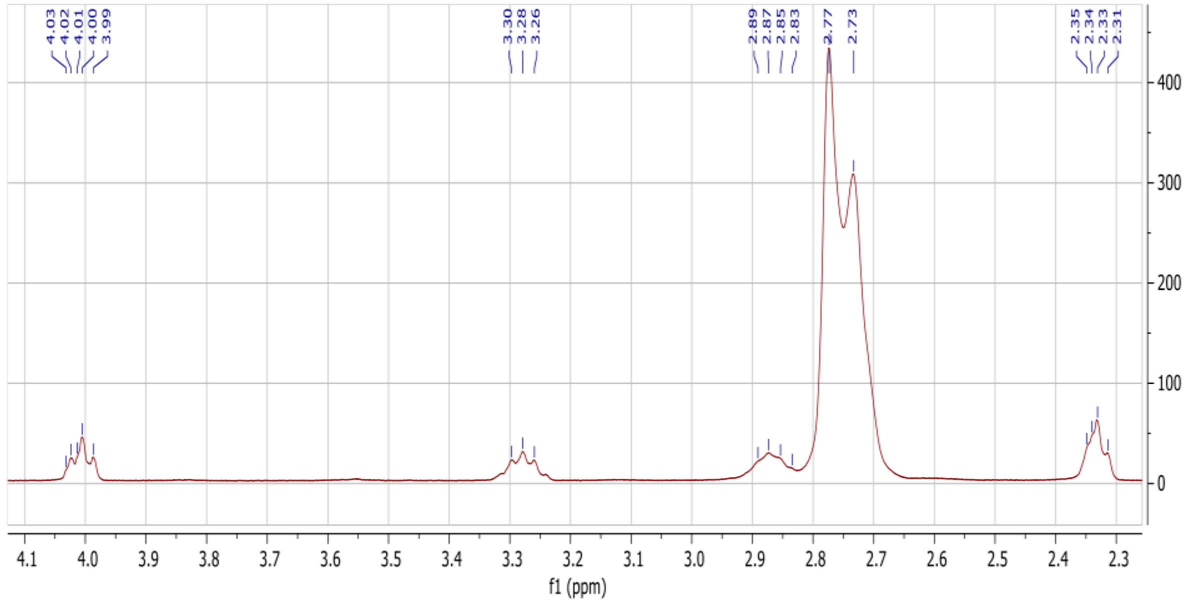

Figure S42. ^{1}H-NMR spectrum (Pyridine-d_5, 500 MHz) of compound **21**

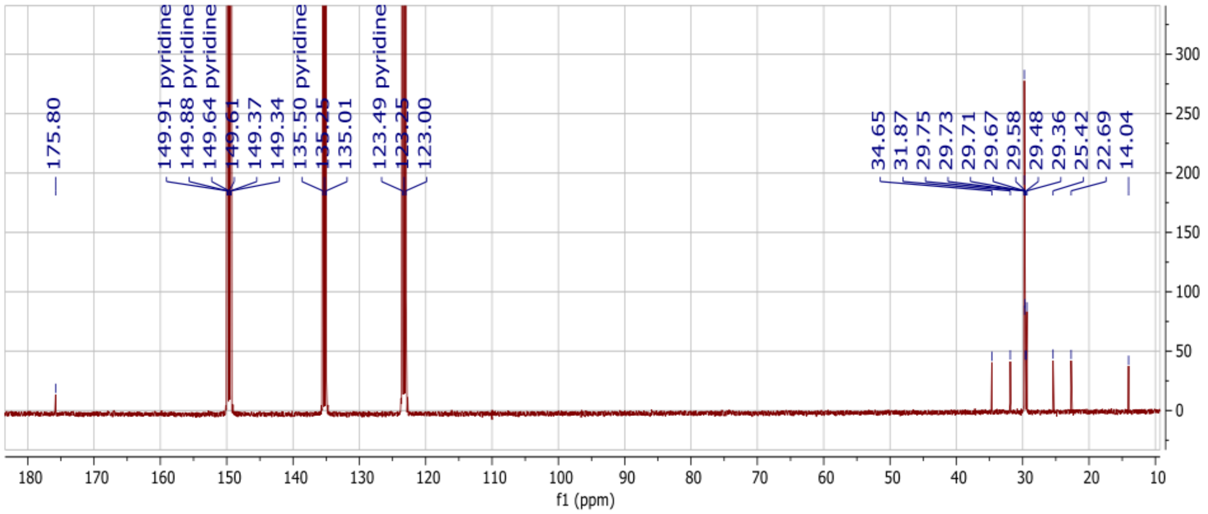

Figure S43. ^{13}C-NMR spectrum (Pyridine-d_5, 125 MHz) of compound **21**

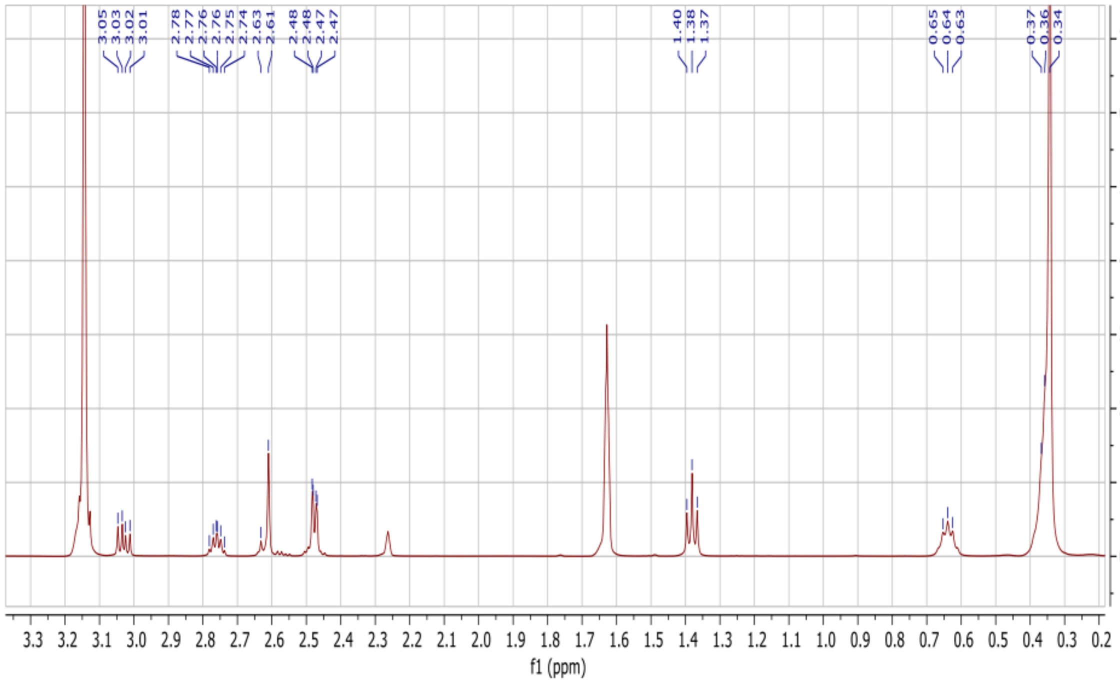

Figure S44. ^{1}H-NMR spectrum (DMSO-d_6, 500 MHz) of compound **22**

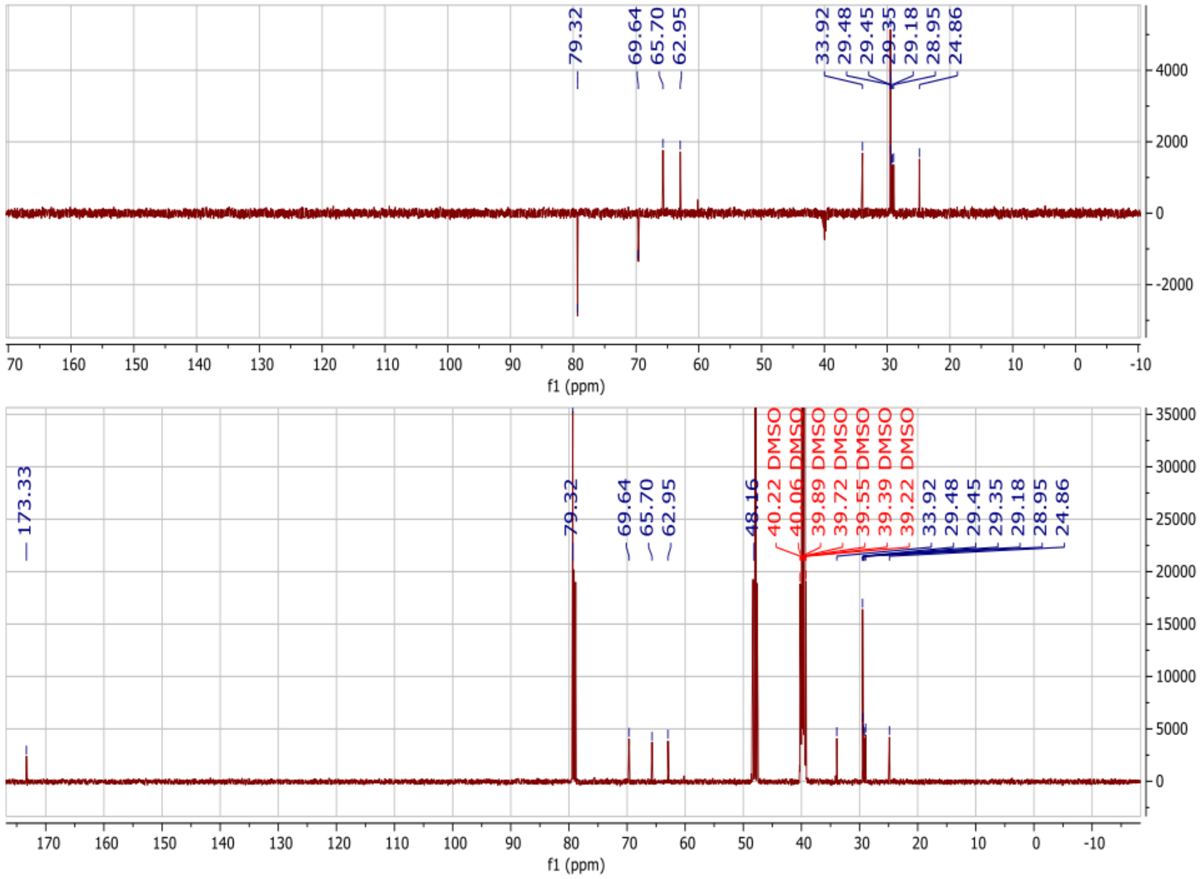

Figure S45. ^{13}C-NMR and DEPT 135 spectra (DMSO-d_6, 125 MHz) of compound **22**
